# Exploring the role of the *CapG* gene in hypoxia adaptation in Tibetan pigs

**DOI:** 10.3389/fgene.2024.1339683

**Published:** 2024-04-12

**Authors:** Feifei Yan, Yu Wang, Mingbang Wei, Jian Zhang, Yourong Ye, Mengqi Duan, Yangzom Chamba, Peng Shang

**Affiliations:** ^1^ College of Animal Science, Tibet Agriculture and Animal Husbandry College, Linzhi, Tibet, China; ^2^ The Provincial and Ministerial Co-Founded Collaborative Innovation Center for R & D in Tibet Characteristic Agricultural and Animal Husbandry Resources, Linzhi, Tibet, China; ^3^ Key Laboratory for the Genetic Improvement and Reproduction Technology of the Tibetan Swine, Linzhi, Tibet, China

**Keywords:** *CapG*, hypoxia adaptation, Tibetan pigs, lung, SNP analysis

## Abstract

**Introduction:** The *CapG* gene, which is an actin-binding protein, is prevalent in eukaryotic cells and is abundantly present in various pathways associated with plateau hypoxia adaptation. Tibetan pigs, which have inhabited high altitudes for extended periods, provide an excellent research population for investigating plateau hypoxia adaptation.

**Results:** This study focused on Tibetan pigs and Yorkshire pigs residing in Nyingchi, Tibet. The blood physiological data of Tibetan pigs were found to be significantly higher than those of Yorkshire pigs, including RBC, HGB, HCT, MCH, and MCHC. The SNP analysis of the *CapG* gene identified six sites with mutations only present in Tibetan pigs. Notably, the transcription factors at sites C-489T, C-274T, and A-212G were found to be altered, and these sites are known to be associated with hypoxia adaptation and blood oxygen transportation. The mRNA expression of the *CapG* gene exhibited highly significant differences in several tissues, with the target proteins predominantly higher in the Yorkshire pig compared to the Tibetan pig. Specifically, a notable difference was observed in the lung tissues. Immunohistochemistry analysis revealed high expression levels of *CapG* proteins in the lung tissues of both Tibetan and Yorkshire pigs, primarily localized in the cytoplasm and cell membrane.

**Conclusion:** The *CapG* gene plays a significant role in regulating hypoxia adaptation in Tibetan pigs. This study provides a theoretical basis for the conservation and utilization of Tibetan pig resources, the breeding of highland breeds, epidemic prevention and control, and holds great importance for the development of the highland livestock economy.

## 1 Introduction

The Tibetan pig is a unique breed in China, specifically adapted to the high-altitude areas of the Qinghai-Tibetan Plateau. This plateau, with an average altitude of 4,000 m, presents a challenging environment characterized by low temperatures, low atmospheric pressure, and hypoxia (Ai et al., 2014; Zhang et al., 2017; Shang et al., 2020). The Tibetan pig has undergone natural selection to survive under these conditions, resulting in distinct physical characteristics such as a small body size, black and long hair, and firm skin. It is typically raised using a semi-housed feeding method that includes fine feed as the primary source and supplemented with wild plants for outdoor intake. These feeding practices contribute to the tender meat texture, firm taste, and agile movement of Tibetan pigs (Shang et al., 2019a; Wang et al., 2021). Consequently, Tibetan pigs have been extensively studied as a significant food source for high-altitude Tibetan communities, given their ability to adapt to the plateau’s hypoxic conditions. The prolonged exposure and evolution in this hypoxic region have contributed to the development of unique plateau characteristics and a stable genetic mechanism for hypoxia adaptation (Zhang et al., 2017; Yuan et al., 2022). Thus, Tibetan pigs are ideal subjects for studying the mechanisms of hypoxia adaptation.

The oxygen concentration in the air varies significantly at different altitudes, with higher altitudes experiencing lower oxygen levels. To prevent excessive harm, organisms undergo specific changes when exposed to this environment. Initially, when an organism first arrives at the plateau, compensatory adjustments occur within the body to adapt to the low-oxygen environment (Shao et al., 2020). Over time, individuals who have lived on the plateau for generations or for an extended period become accustomed to this low-oxygen environment, a process known as low-oxygen adaptation (Hochachka et al., 1998). Hypoxia adaptation, in essence, entails efficiently utilizing limited oxygen to ensure normal physiological functions under hypoxic conditions. In the low-oxygen environment of the plateau, various reactions occur during oxygen utilization, including uptake and internal transport of oxygen from the environment, oxygen transportation and exchange in the blood, cellular oxidation, and ultimately achieving a balance and hypoxic adaptation. Through genetic preservation, animals adapted to chronic permanent hypoxia maintain homeostasis (Eaton and Pamenter, 2022). Ramirez et al. (2007) discovered that most animals respond to reduced oxygen availability by reducing their energy metabolism within a low-oxygen environment (Cho et al., 2021). However, reducing energy metabolism is not suitable for all species, such as birds with increased oxygen uptake during flight, requiring heightened oxygen transport capacity (Storz et al., 2010). Hsia et al. (2007) demonstrated that moving foxhounds to high-altitude areas enhanced their lung’s oxygen dispersion ability. Similarly, Hsia et al. (2005) compared guinea pig lung morphology at different altitudes and found that at higher altitudes, the alveoli and lung size were larger. Recent studies have revealed that hypoxic adaptation involves multiple molecular mechanisms that regulate not only oxygen transport but also oxygen utilization in cellular metabolism (Murray, 2016). At the cellular level, the hypoxia-inducible factor (HIF) pathway mediates the transcriptional response to hypoxia (Shang et al., 2019b), promoting glycolysis and inhibiting oxidative metabolism (Chen and Gaber, 2021; Malkov et al., 2021; Batie et al., 2022). It has been observed that species lacking hypoxia adaptation experience reduced oxygen metabolism and increased production of reactive oxygen species (ROS) in the mitochondria, leading to oxidative damage. Finally, it should be noted that ROS plays a vital role in fundamental biological processes such as cell proliferation, differentiation, and cell death pathways (Mittler, 2017; McGarry et al., 2018).

Zhang et al. (2017) conducted a study in 2017 where they analyzed transcriptomic and proteomic data from heart tissues of Tibetan and Yorkshire pigs reared in highland and lowland areas. They found significant differences in genes and pathways related to hypoxic adaptation, including the *HIF-1* and *VEGF* signaling pathways. Ai et al. (2014) performed a genome-wide analysis on Tibetan pig populations and lowland breeds, identifying genes that have undergone positive selection in Tibetan pigs. Li et al. (2013) analyzed the genetic mechanism, population structure, and evolutionary model of Tibetan pigs, identifying genes and pathways involved in hypoxic adaptation. *CapG*, also known as macrophage-capping protein (MCP), is an actin-binding protein found in various tissues and expressed in eukaryotic cells. It plays a role in cell signaling, phagocytosis, cellular transport, and other cellular mechanisms (Gurtu and Michelakis, 2015). *CapG* proteins regulate cell motility, development, maintenance of cell morphology, and intracellular calcium and phosphatidylinositol levels (Jurasz et al., 2010). The rapid growth of protein filaments is involved in actin-based cell motility and membrane ruffling (Silacci et al., 2004). In a proteomics study, HIF-1 was found to bind to a hypoxia-responsive element in the *CapG* gene, which is critical for the expression of HIF-1-induced genes. The expression of *CapG* was also observed to increase in lung cancer cell lines under hypoxic conditions (Shao et al., 2011). In a screening study of candidate genes related to hypoxia adaptation, the *CapG* gene was found to be expressed in both Tibetan and Yorkshire pigs and enriched in regulatory pathways related to plateau hypoxia acclimatization (Shang et al., 2022).

This study aims to analyze the *CapG* gene at the DNA, mRNA, protein, and phenotype levels in Tibetan pigs to understand the mechanism of hypoxia adaptation in plateau animals. The findings will contribute to our understanding of molecular mechanisms of animal adaptation to the plateau environment and genetics. They will also provide a basis for the preservation and utilization of Tibetan pig germplasm resources and breeding of plateau varieties, benefiting the plateau animal husbandry industry. Tibetan pigs, having adapted to high altitudes, are ideal subjects for studying plateau hypoxia adaptation.

## 2 Materials and methods

### 2.1 Animals and groups

This study examined Tibetan pigs from the teaching practice farm of the School of Animal Science at the Tibetan College of Agriculture and Animal Husbandry, as well as Yorkshire pigs bred at Nyingchi Yugao Ecological Agricultural Development Co. The pigs are fed on an antibiotic-free diet and housed according to routine procedures. They are also regularly vaccinated and wormed, and all pigs have free access to food and water. Blood samples were collected from each pig using a 10 mL syringe via the anterior vena cava. The samples were swiftly returned to the laboratory for assessment of physiological parameters. Randomly selected ear tissue samples were obtained from 50 Tibetan pigs and 50 Yorkshire pigs and placed in 2 mL centrifuge tubes containing 75% alcohol. The samples were then stored at a lower temperature for the DNA extraction process. Heart, liver, spleen, lung, and kidney tissues were collected and placed in tubes filled with RNA preservation solution. The lung tissue samples were preserved using liquid nitrogen and transported at −80°C to the laboratory for RNA extraction. During the collection phase, 3 cm^3^ tissue samples were fixed in a 4% neutral formaldehyde solution to retain their structural integrity, with care taken to avoid sample extrusion. All the research work was carried out in strict accordance with the guidelines approved by the Animal Welfare Committee of Tibet Agriculture and Animal Husbandry University.

### 2.2 Blood routine examination

Prior to performing physiological index analysis using an automatic animal blood cell analyzer, the collection tube containing sodium heparin anticoagulant was gently inverted. The measured parameters included White Blood Cell Count: WBC (×10^9^/L), Red Blood Cell Count: RBC (×10^12^/L), Hemoglobin: HGB (g/L), Hematocrit: HCT (%), Mean Corpuscular Volume: MCV (fL), Mean Corpuscular Hemoglobin: MCH (pg), Mean Corpuscular Hemoglobin Concentration: MCHC (g/L), Red Cell Distribution Width: RDW (%), Platelet Count: PLT (×10^9^/L), Mean Platelet Volume: MPV (fL), Platelet Volume Distribution Width: PDW (fL), and Procalcitonin: PCT (%).

### 2.3 SNP screening and genotyping

Genomic DNA was extracted from pig ear tissue using the phenol-chloroform technique. The DNA sequence of the *sus scrofa CapG* gene (accession number: NC_010445.4) was retrieved from GenBank (www.ncbi.nlm.nih.gov/). Primer design was conducted using Primer 5.0 software, based on the 2 kb region upstream of the respective start codon and the coding sequence (CDS) region of this gene. Amplification of the 5′ flanking region and coding sequence (CDS) of the *CapG* gene was performed using ear tissue samples obtained from pigs (see Schedules 1, 2). Single nucleotide polymorphisms (SNPs) were identified using Chromas Pro software, and the corresponding gene and genotype frequencies were calculated based on the sequencing results. The mutated loci were analyzed for transcription factor binding sites using the website https://jaspar.elixir.no/ before and after mutation.

**SCHEDULE 1:** CapG gene primer information.

Name of primers	Primer sequences (5′-3′)	Annealing temperature	Size
1:5′-*CapG-*(1)-3′	F:CCATCCTGGTCTATCCTCG	58°C	768bp
	R:AGTGGACCCAAGCAGAGAC		
2:5′-*CapG-*(2)-3′	F:CTCAGCCCCCAATAGTCTCC	58°C	909bp
	R:CAGACTTGTGGGTGCCGA		
3:5′-*CapG-*(3)-3′	F:AGAGAGATGAGGGACGGG	60°C	799bp
	R:CAGCAGTGAGTGCGAGAGA		
4:5′-CDS-*CapG*-(1)-3′	F:CGTGAGAACCAGGGCATCTT	62°C	952bp
	R:CCCACCCTCATTTCCAGTCC		
5:5′-CDS-*CapG*-(2)-3′	F:CCACCTGCACCTGTGGATAG	60°C	784bp
	R:GCGGGAGATGAAGTCCTCAG		

**SCHEDULE 2:** CapG gene quantitative primer information.

Login number	Gene	Primer sequences (5′-3′)	Size
XM_021087297	*CapG*	F:CCTGGAGCGTAACAAAGCG	192bp
		R:GCCTGGGCATTTTTCTGGT	
XM_021086047	*β-actin*	F:TCTGGCACCACACCTTCTA	127bp
		R:AAGGTCTCGAACATGATCTG	

### 2.4 RT-qPCR

To perform real-time fluorescence quantitative PCR, RNA from different tissues of Tibetan and Yorkshire pigs was extracted using a Total RNA extraction kit. The mRNA sequence of the pig *CapG* gene (Login number: XM_021087297) was downloaded from the NCBI website. β-actin (Login number: XM_021086047) was selected as the internal reference gene. Primer design was done using Primer5.0, and the primers were synthesized by Sangon Biotech (Shanghai) Co., Ltd. Three replicates of each sample were prepared for RT-qPCR, and the relative mRNA expression of the *CapG* gene was calculated using the 2^−ΔΔCT^ method.

### 2.5 Western blot

For the Western blot analysis, protein tissues from both Tibetan and Yorkshire pigs were extracted. A polyacrylamide gel was prepared, and the quantity of protein samples was determined based on the measured protein concentration. The denatured samples were placed in the gel, and the electrophoresis apparatus was set up. The gel was washed with water and then prepared for membrane transfer by adding the fiber mat, filter paper, and pre-soaked PVDF membrane (in methanol) to the membrane transfer solution for 5–10 min. These were added to the membrane transfer folder in a specific order. The molds, along with the membrane transfer liquid, were placed into the membrane transfer instrument, and the molds were transferred accordingly. The PVDF membrane was inserted into the rapid membrane transfer solution provided by Suzhou New Saimei Biotechnology Co., Ltd., and incubated in a shaker at 37°C for 30 min. The sealed PVDF membrane was washed with PBS, followed by incubation with the primary antibody (diluted 1–2000: Shanghai Povan Biotechnology Co., Ltd.) overnight at 4°C. The membrane was then washed three times for 10 min each with 1x TBST solution on a horizontal shaker. Subsequently, the washed PVDF membrane and secondary antibody (1:10,000 dilution from ImmunoWay Biotechnology Company) were incubated in a self-sealing pouch at 37°C for 1 h on a shaker. The membrane was again washed three times for 10 min each with 1x TBST solution on a horizontal shaker. Finally, the washed PVDF membrane was incubated at 37°C for 1 h in a self-sealing bag with ECL from Beijing CryptoWay Biotechnology Company. The membranes underwent three washes with 1x TBST solution using a horizontal shaker for 10 min each.

### 2.6 Immunohistochemical analysis

The lung tissue samples were prepared for immunohistochemical analysis by fixing them and cutting them into 0.5 cm^3^ pieces using a scalpel blade. These samples were then placed into disposable embedding boxes for further processing. The sections were dewaxed and washed using varying concentrations of anhydrous ethanol and PBS. This was done to ensure proper cleaning of the tissue samples before the immunohistochemistry experiments. For the immunohistochemistry experiments, the SP Detection Kit from Beijing Solepol Technology Co., Ltd. was used. The experiments were conducted strictly following the instruction manual provided with the kit. This ensured accurate and reliable results. After the immunohistochemistry experiments, the sections were stained using hematoxylin for chromogenic analysis. They were then sealed with neutral gum to preserve the staining and prevent any damage to the sections. To analyze the results, the grey density method was employed. This involved analyzing the sections using a technique that measures the intensity of the staining and converts it into grey density values.

### 2.7 Statistical analyses

For statistical analysis, the mutation sites were analyzed using Chromas Pro sequencing results. The relevant data underwent statistical analysis using IBM SPSS Statistics 26.0. Significance was determined based on the probability values (*p*-values). A *p*-value less than 0.05 or 0.01 was considered highly significant. To conduct the grey density analysis, ImageJ software was used.

## 3 Results

### 3.1 Analysis of blood physiological indices

Table 1 presents the blood physiological parameters of Tibetan pigs and Yorkshire pigs. Tibetan pigs demonstrated significantly higher levels of RBC, HGB, and HCT compared to Yorkshire pigs, with a *p*-value of less than 0.01. Additionally, Tibetan pigs exhibited significantly higher values of MCH and MCHC (*p* < 0.05), whereas Yorkshire pigs had significantly greater RDW values than Tibetan pigs (*p* < 0.01). However, no significant difference (*p* > 0.05) was observed in the other physiological indices between the two species.

**TABLE 1:** Blood physiological index data.

Index	Tibetan pigs	Yorkshire pigs
WBC (×10^9^/L)	32.98 ± 8.32	28.40 ± 8.14
RBC (×10^12^/L)	8.51 ± 0.54^∗∗^	6.86 ± 0.66
HGB (g/L)	193.40 ± 16.73^∗∗^	151.8 ± 24.28
HCT (%)	55.18 ± 4.05^∗∗^	44.32 ± 6.27
MCV (fL)	67.20 ± 3.55	64.58 ± 6.25
MCH (pg)	23.30 ± 1.54^∗^	22.04 ± 2.65
MCHC (g/L)	350.48 ± 10.10^∗^	341.64 ± 15.01
RDW (%)	13.36 ± 0.89^∗∗^	14.79 ± 1.38
PLT (×10^9^/L)	272.80 ± 64.59	242.36 ± 96.65
MPV (fL)	7.81 ± 0.66	8.07 ± 1.17
PDW (fL)	18.30 ± 2.21	18.34 ± 1.06
PCT (%)	0.21 ± 0.06	0.20 ± 0.07

### 3.2 SNPs loci in Tibetan and Yorkshire pig

**FIGURE 1:**
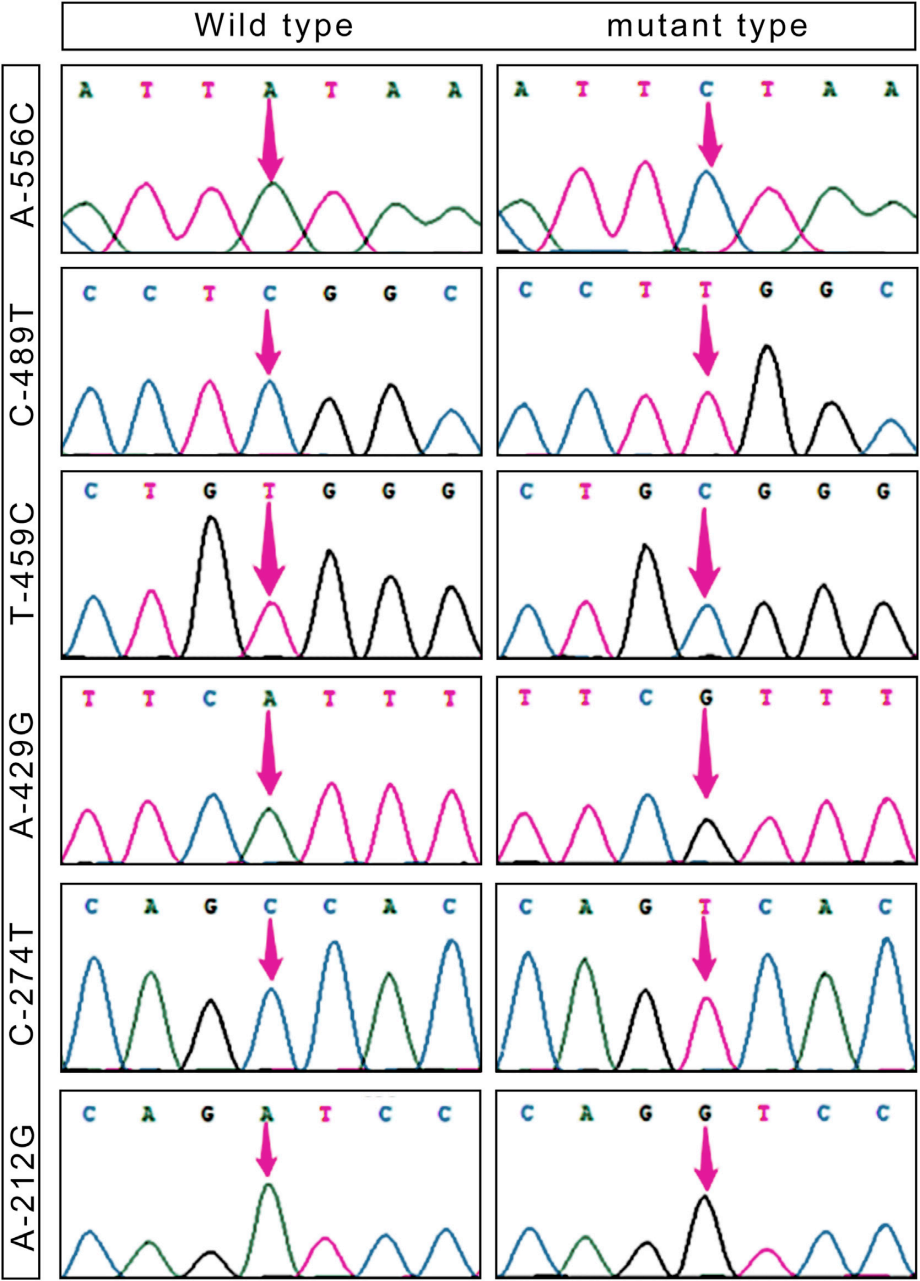
SNPs loci in the 5′ flanking region of the *CapG* gene in Tibetan pigs.

Based on the sequencing results, it has been determined that the 5′ flanking area of only the *CapG* gene of Tibetan pigs exhibits six mutation sites: A-556C, C-489T, T-459C, A-429G, C-274T, and A-212G (Figure 1). Notably, these mutations are not present in Yorkshire pigs.

Table 2 presents the six single nucleotide polymorphism (SNPs) loci observed in Tibetan pigs. These include an A/C mutation at −556 bp from the start codon, designated as A-556C, a C/T mutation at −489 bp, designated as C-489T, a T/C mutation at −459 bp, designated as T-459C, an A/G mutation at −429 bp, designated as A-429G, a C/T mutation at −274 bp, designated as C-274T, and an A/G mutation at −212 bp, designated as A-212G. All six mutation sites underwent rigorous testing and were determined to conform to the Hardy-Weinberg equilibrium (*p* > 0.05).

**TABLE 2:** Genotype frequencies and chi-square test of SNPs loci for *CapG* gene.

Site	From	Genotype frequency			Chi-square (*p*-value)	Frequency of allele genotypes	
		AA	AC	CC		A	C
A-556C	Tibetan pigs (TP)	14/0.35	0/0.00	26/0.65	1.31 (0.52)	0.35	0.65
	Yorkshire pig (YY)	41/1.00	0/0.00	0/0.00	—	1	0.00
	TP vs. YY	χ^2^ = 39.248 *p* = 3.7322E-10					
		CC	CT	TT		C	T
C-489T	Tibetan pigs (TP)	14/0.35	0/0.00	26/0.65	1.31 (0.52)	0.35	0.65
	Yorkshire pig (YY)	41/1.00	0/0.00	0/0.00	—	1	0.00
	TP vs. YY	χ^2^ = 39.248 *p* = 3.7322E-10					
		TT	TC	CC		T	C
T-459C	Tibetan pigs (TP)	14/0.35	0/0.00	26/0.65	1.31 (0.52)	0.35	0.65
	Yorkshire pig (YY)	41/1.00	0/0.00	0/0.00	—	1	0.00
	TP vs. YY	χ^2^ = 39.248 *p* = 3.7322E-10					
		AA	AG	GG		A	G
A-429G	Tibetan pigs (TP)	14/0.35	0/0.00	26/0.65	1.31 (0.52)	0.35	0.65
	Yorkshire pig (YY)	41/1.00	0/0.00	0/0.00	—	1	0.00
	TP vs. YY	χ^2^ = 39.248 *p* = 3.7322E-10					
		CC	CT	TT		C	T
C-274T	Tibetan pigs (TP)	14/0.35	0/0.00	26/0.65	1.31 (0.52)	0.35	0.65
	Yorkshire pig (YY)	41/1.00	0/0.00	0/0.00	—	1	0.00
	TP vs. YY	χ^2^ = 39.248 *p* = 3.7322E-10					
		AA	AG	GG		A	G
A-212G	Tibetan pigs (TP)	14/0.35	0/0.00	26/0.65	1.31 (0.52)	0.35	0.65
	Yorkshire pig (YY)	41/1.00	0/0.00	0/0.00	—	1	0.00
	TP vs. YY	χ^2^ = 39.248 *p* = 3.7322E-10					

Transcription factor prediction was conducted at the six mutation sites. It was determined that there were no alterations to transcription factors preceding or following the mutation at A-556C, T-459C, and A-429G. However, the mutation at sites C-489T, C-274T, and A-212G resulted in the emergence of new transcription factors, namely, KLF4, GFI1, and RUNX1.

### 3.3 Tibetan and Yorkshire pigs: *CapG* gene expression

**FIGURE 2:**
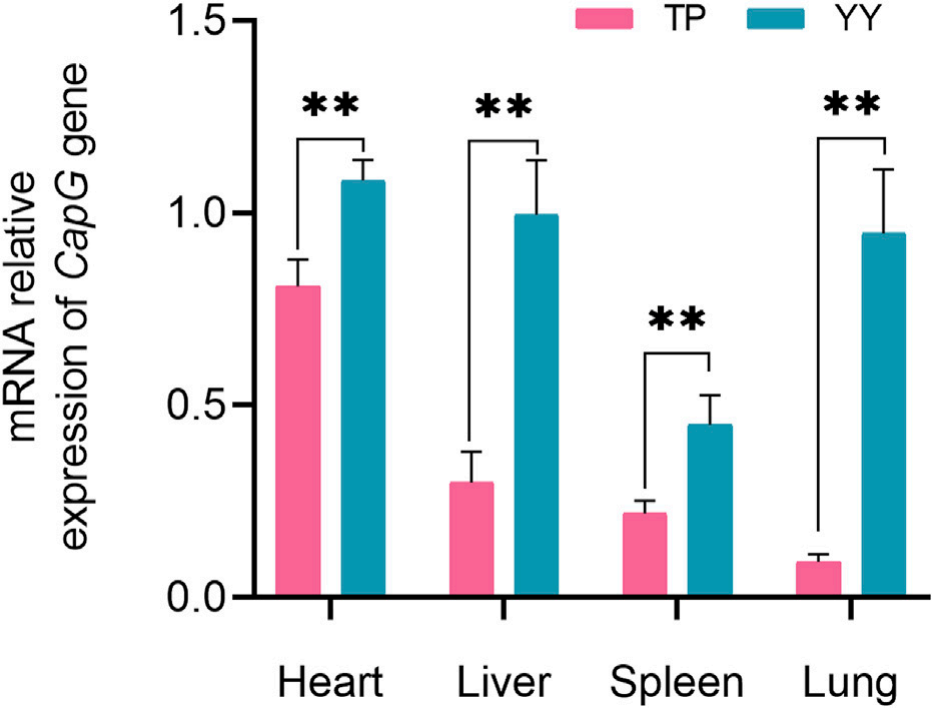
Relative mRNA expression of *CapG* gene in different tissues. TP indicates Tibetan pig and YY indicates Yorkshire pig. ** denotes a highly significant difference with *p* < 0.01.

Comparison of *CapG* Gene Expression in Tibetan and Yorkshire Pigs, Figure 2 depicts the mRNA expression levels of the *CapG* gene in the heart, liver, spleen, and lungs of Tibetan and Yorkshire pigs. The analysis revealed a significant increase in the relative expression of the *CapG* gene in Yorkshire pigs compared to Tibetan pigs (*p* < 0.01).

### 3.4 Expression of *CapG* proteins in tissues

**FIGURE 3:**
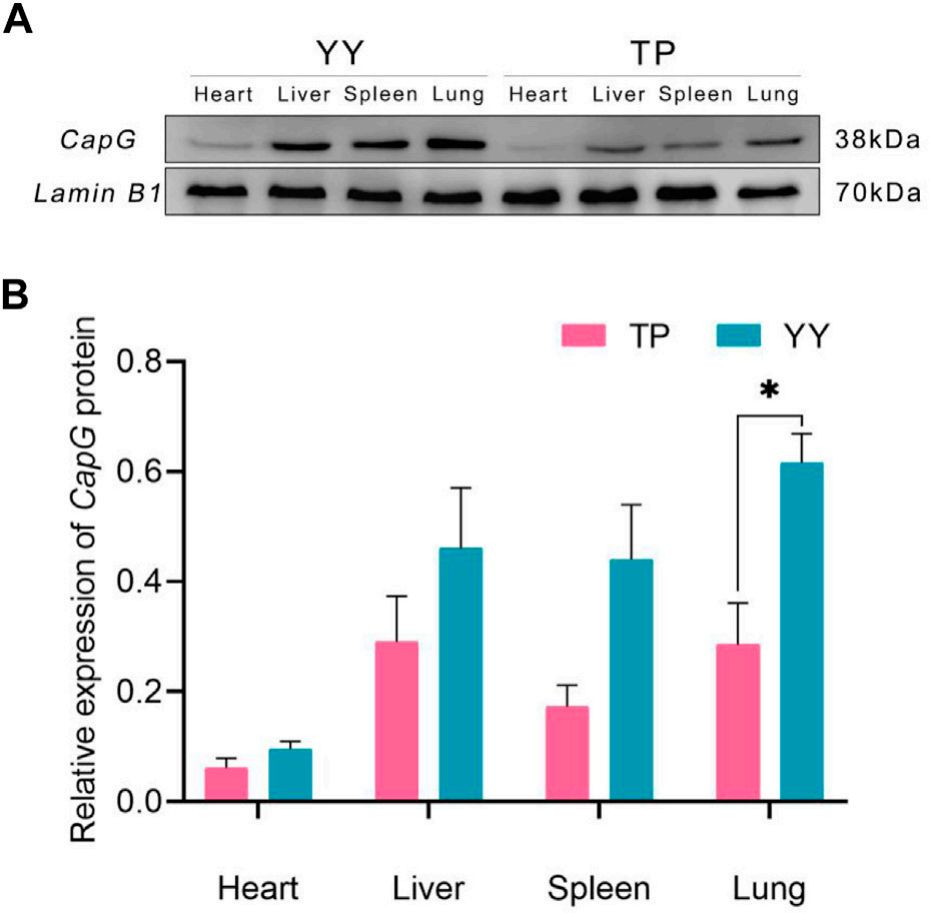
Relative expression of *CapG* protein in different tissues. TP indicates Tibetan pig and YY indicates Yorkshire pig. **(A)** Protein bands of *CapG* protein in different tissues; **(B)** Relative expression of *CapG* protein in different tissues.

Figure 3 demonstrates that there were no statistically significant differences (*p* > 0.05) in *CapG* protein expression levels in the heart, liver, and spleen between Tibetan and Yorkshire pigs. However, a general trend indicates that Yorkshire pigs tended to exhibit higher expression levels compared to Tibetan pigs. Notably, in lung tissues, the data revealed a significant increase in *CapG* protein expression in Yorkshire pigs compared to Tibetan pigs (*p* < 0.05).

**FIGURE 4:**
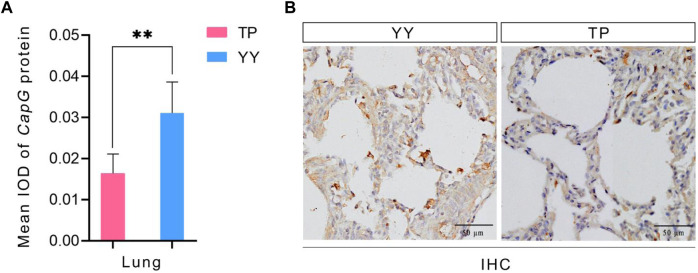
IHC and mean IOD values of different pig *CapG* proteins. TP indicates Tibetan pig and YY indicates Yorkshire pig. **(A)** Mean IOD of *CapG* protein; **(B)** IHC of different pig lung tissue.

The immunohistochemical results of Tibetan and Yorkshire pigs, evaluated using ImageJ software, are presented in Figure 4A. We observed significantly higher mean Integrated Optical Density (IOD) values for *CapG* protein in the lung tissue of Yorkshire pigs compared to Tibetan pigs (*p* < 0.01). As shown in Figure 4B, the lung tissue of Yorkshire pigs displayed prominent brownish-yellow and yellow staining, indicating a strong positive reaction. In contrast, the lung tissue of Tibetan pigs exhibited a weaker positive reaction and lighter color. Extensive expression of *CapG* protein was observed in the lung tissue of both Tibetan and Yorkshire pigs, with the most intense expression observed in the cell membrane and cytoplasm.

## 4 Discussion

Tibetan pigs exhibited significantly higher indices for RBC, HGB, HCT, MCH, and MCHC compared to Yorkshire pigs, while the RDW was significantly higher in Yorkshire pigs. These findings indicate that Tibetan pigs possess a superior capacity for oxygen transport, which is a physiological manifestation of their long-term adaptation to a plateau environment and contributes to their improved ability to tolerate hypoxia (Wang et al., 2017). A higher number of RBC and HGB results in a larger surface area of the blood red cell membrane and greater oxygen-carrying capacity, promoting efficient gas exchange between hemoglobin in red cells and tissue cells, thereby enhancing the rate of blood oxygen metabolism. Increased RBC and HGB numbers in the blood result in a larger surface area of the erythrocyte membrane, enhancing the oxygen-carrying capacity and facilitating efficient gas exchange between hemoglobin in erythrocytes and tissue cells, thereby enhancing the rate of oxygen metabolism. Additionally, erythrocyte count is associated with the capacity for carrying both oxygen and carbon dioxide, as well as immune function. Consistently, Tibetan pigs have adapted to the low oxygen conditions of the plateau by increasing their RBC, HGB, and HCT levels. Notably, high-altitude species with robust low oxygen adaptation exhibit significantly higher blood oxygen transport and release capacity compared to corresponding lowland species.

Plateau hypoxia leads to a range of heritable physiological changes, including hematological indices, energy metabolism, transcriptional regulation, and antioxidant capacity. Yi et al. (2010), Arciero et al. (2018), and Zhang et al. (2019) conducted a genome-wide scanning of polymorphic sites in EPAS1, EGLN1, RUVBL1, and SLC4A3 genes, establishing their association with hypoxic adaptation. In their experiments on a mouse model of pulmonary arterial hypertension, Xu et al. (2016) demonstrated that the CapG gene is involved in the remodelling mechanism of the pulmonary vasculature in mice with pulmonary arterial hypertension and is associated with the proliferation of pulmonary artery smooth muscle cells. The *CapG* gene was examined in this study to identify pig polymorphic sites at the DNA level. A one-generation sequencing and screening approach was employed to investigate mutation sites in the 2 kb region upstream of the start codon and CDS region of the gene. The results revealed six mutation sites, namely, A-556C, C-489T, T-459C, A-429G, C-274T, and A-212G, in the 5′ flanking region of the *CapG* gene in Tibetan pigs. Importantly, all of these mutation sites were consistent with the Hardy-Weinberg equilibrium. Transcription factor prediction revealed the presence of three specific transcription factors at different loci: KLF4 at the C-489T locus associated with hypoxic adaptive capacity (Sangwung et al., 2017), GFI1 at the C-274T locus associated with hem oxygen transport (Kang et al., 2022), and RUNX1 at the A-212G locus, involved in regulating the response to hypoxia (Gao et al., 2018). These findings provide insights into potential mechanisms of transcriptional regulation in response to hypoxia. Krüppel-like factor 4 (KLF4) belongs to the KLF family of transcription factors and is associated with cell proliferation, differentiation, apoptosis, as well as promoting pulmonary angiogenesis and blood transport. KLF4 facilitates oxygen transport and improves the lung’s hypoxic status (Ghaleb and Yang, 2017). Additionally, KLF4 and EGR1 genes may jointly regulate BCL6B genes, forming the KLF4-EGR1-BCL6B complex. The KLF4 gene likely acts as the main regulator of the KLF4-EGR1-BCL6B complex, enabling the TGF-β signaling pathway and enhancing hypoxia resistance in Tibetan pigs. Furthermore, RUNT-related transcription factor 1 (RUNX1) plays a crucial role in regulating hematopoietic stem cell differentiation (Kamikubo, 2018). Zhang and Chen (2009) proposed that RUNX1 interacts with HIF-1 to modulate its transcriptional activity. Therefore, these three loci are considered pivotal in regulating the hypoxia adaptation mechanism in Tibetan swine.

The expression of the *CapG* gene was significantly higher in the heart, liver, spleen, and lung tissues of Yorkshire pigs compared to Tibetan pigs (*p* < 0.01). The findings are in line with Xu et al. (2016) study, which showed that the CapG gene is preferentially expressed in pulmonary artery smooth muscle cells under hypoxic conditions. This breed-specific difference in expression levels may be attributed to the prolonged exposure of Tibetan pigs to the hypoxic conditions of the plateau over generations, resulting in the development of a stable plateau adaptation mechanism (Zhang et al., 2009). In contrast, Yorkshire pigs, being an exotic species, exhibit increased expression of the *CapG* gene under hypoxic conditions, potentially to alleviate pulmonary arterial hypertension and adapt to the hypoxic environment. Hence, Tibetan pigs demonstrate greater adaptability to hypoxia than Yorkshire pigs (Xu et al., 2016). Immunohistochemical analysis revealed high expression of the *CapG* protein in the lung tissues of both Tibetan and Yorkshire pigs, primarily located in the cytoplasm and cell membrane. However, the expression level of *CapG* protein was significantly higher in the lungs of Yorkshire pigs compared to Tibetan pigs (*p* < 0.01). These results confirm the distinctive expression pattern of *CapG* protein, particularly its high expression in the lung tissues of Yorkshire pigs (*p* < 0.05). In the heart, liver, and spleen tissues, the expression of *CapG* protein was higher in Yorkshire pigs compared to Tibetan pigs, although the difference was not statistically significant (*p* > 0.05). The regulation of actin-based cell movement in muscle cells is controlled by *CapG* protein (Cunningham et al., 1991). Overexpression of this protein in fibroblasts and endothelial cells can enhance cellular activity (Sun et al., 1995). *CapG* protein facilitates cytoskeletal filament formation and actin assembly through its interaction with actin. Additionally, it plays a crucial role in regulating vital cellular activities such as cell differentiation, phagocytosis, and cell migration (Chi et al., 2019). A comparison between the protein-level results and mRNA level results showed consistent expression of *CapG* in both cases. These findings further emphasize the important role of *CapG* in hypoxia adaptation in pigs and provide valuable insights into the regulatory mechanisms underlying hypoxia adaptation through *CapG*.

## 5 Conclusion

The blood physiological indices of Tibetan and Yorkshire pigs were examined in this research. The findings revealed that Tibetan pigs had significantly higher values for five indices compared to Yorkshire pigs. Furthermore, transcription factor prediction analysis identified the emergence of new factors after mutation, all of which were associated with hypoxia regulation. The results obtained from RT-qPCR and *CapG* protein analysis consistently supported the role of *CapG* protein in regulating hypoxia adaptation mechanisms. Overall, Tibetan pigs demonstrated impressive blood oxygen transportation capacity, with the *CapG* gene being a significant candidate gene involved in regulating hypoxia adaptation in this species. Notably, this gene co-regulates hypoxia adaptation mechanisms in Tibetan pigs at multiple levels.

## Data Availability

The datasets presented in this study can be found in online repositories. The names of the repository/repositories and accession number(s) can be found in the article/Supplementary Material.

## References

[B1] Ai H. Yang B. Li J. Xie X. Chen H. Ren J. (2014). Population history and genomic signatures for high-altitude adaptation in Tibetan pigs. BMC genomics 15 (1), 834. 10.1186/1471-2164-15-834 25270331 PMC4197311

[B2] Arciero E. Kraaijenbrink T. Haber M. Mezzavilla M. Ayub Q. Wang W. (2018). Demographic history and genetic adaptation in the himalayan region inferred from genome-wide SNP genotypes of 49 populations. Mol. Biol. Evol. 35 (8), 1916–1933. 10.1093/molbev/msy094 29796643 PMC6063301

[B3] Batie M. Frost J. Shakir D. Rocha S. (2022). Regulation of chromatin accessibility by hypoxia and HIF. Biochem. J. 479 (6), 767–786. 10.1042/BCJ20220008 35258521 PMC9022986

[B4] Chen Y. Gaber T. (2021). Hypoxia/HIF modulates immune responses. Biomedicines 9 (3), 260. 10.3390/biomedicines9030260 33808042 PMC8000289

[B5] Chi Y. Xue J. Huang S. Xiu B. Su Y. Wang W. (2019). CapG promotes resistance to paclitaxel in breast cancer through transactivation of PIK3R1/P50. Theranostics 9 (23), 6840–6855. 10.7150/thno.36338 31660072 PMC6815964

[B6] Cho H. Loreti E. Shih M. Perata P. (2021). Energy and sugar signaling during hypoxia. New phytologist 229 (1), 57–63. 10.1111/nph.16326 31733144

[B7] Cunningham C. Stossel T. Kwiatkowski D. (1991). Enhanced motility in NIH 3T3 fibroblasts that overexpress gelsolin. Sci. (New York, N.Y.) 251 (4998), 1233–1236. 10.1126/science.1848726 1848726

[B8] Eaton L. Pamenter M. E. (2022). What to do with low O2: redox adaptations in vertebrates native to hypoxic environments. Comp. Biochem. Physiology Part A Mol. Integr. Physiology 271, 111259. 10.1016/j.cbpa.2022.111259 35724954

[B9] Gao L. Tober J. Gao P. Chen C. Tan K. Speck N. (2018). RUNX1 and the endothelial origin of blood. Exp. Hematol. 68, 2–9. 10.1016/j.exphem.2018.10.009 30391350 PMC6494457

[B10] Ghaleb A. Yang V. (2017). Krüppel-like factor 4 (KLF4): what we currently know. Gene 611, 27–37. 10.1016/j.gene.2017.02.025 28237823 PMC5391259

[B11] Gurtu V. Michelakis E. (2015). Cell-based gene therapy in pulmonary arterial hypertension: journeys in translational medicine. Circulation Res. 117 (7), 596–598. 10.1161/CIRCRESAHA.115.307247 26358109

[B12] Hochachka P. Gunga H. Kirsch K. (1998). Our ancestral physiological phenotype: an adaptation for hypoxia tolerance and for endurance performance? Proc. Natl. Acad. Sci. U. S. A. 95 (4), 1915–1920. 10.1073/pnas.95.4.1915 9465117 PMC19213

[B13] Hsia C. C. W. Carbayo J. J. P. Yan X. Bellotto D. J. (2005). Enhanced alveolar growth and remodeling in Guinea pigs raised at high altitude. Respir. Physiology Neurobiol. 147 (1), 105–115. 10.1016/j.resp.2005.02.001 15848128

[B14] Hsia C. C. W. Robert L. Johnson J. McDonough P. Dane D. M. Hurst M. D. (2007). Residence at 3,800-m altitude for 5 mo in growing dogs enhances lung diffusing capacity for oxygen that persists at least 2.5 years. J. Appl. Physiology 102 (4), 1448–1455. 10.1152/japplphysiol.00971.2006 17218427

[B15] Jurasz P. Courtman D. Babaie S. Stewart D. (2010). Role of apoptosis in pulmonary hypertension: from experimental models to clinical trials. Pharmacol. Ther. 126 (1), 1–8. 10.1016/j.pharmthera.2009.12.006 20117135

[B16] Kamikubo Y. (2018). Genetic compensation of RUNX family transcription factors in leukemia. Cancer Sci. 109 (8), 2358–2363. 10.1111/cas.13664 29883054 PMC6113440

[B17] Kang B. Zhang T. Huang K. Wang T. Li Y. Mai Y. (2022). GFI1 regulates chromatin state essential in human endothelial-to-haematopoietic transition. Cell Prolif. 55 (5), e13244. 10.1111/cpr.13244 35504619 PMC9136496

[B18] Li M. Tian S. Jin L. Zhou G. Li Y. Zhang Y. (2013). Genomic analyses identify distinct patterns of selection in domesticated pigs and Tibetan wild boars. Nat. Genet. 45 (12), 1431–1438. 10.1038/ng.2811 24162736

[B19] Malkov M. Lee C. Taylor C. (2021). Regulation of the hypoxia-inducible factor (HIF) by pro-inflammatory cytokines. Cells 10 (9), 2340. 10.3390/cells10092340 34571989 PMC8466990

[B20] McGarry T. Biniecka M. Veale D. Fearon U. (2018). Hypoxia, oxidative stress and inflammation. Free Radic. Biol. Med. 125, 15–24. 10.1016/j.freeradbiomed.2018.03.042 29601945

[B21] Mittler R. (2017). ROS are good. Trends plant Sci. 22 (1), 11–19. 10.1016/j.tplants.2016.08.002 27666517

[B22] Murray A. (2016). Energy metabolism and the high-altitude environment. Exp. Physiol. 101 (1), 23–27. 10.1113/EP085317 26315373

[B23] Ramirez J.-M. Folkow L. P. Blix A. S. (2007). Hypoxia tolerance in mammals and birds: from the wilderness to the clinic. Annu. Rev. Physiology 69 (1), 113–143. 10.1146/annurev.physiol.69.031905.163111 17037981

[B24] Sangwung P. Zhou G. Nayak L. Chan E. Kumar S. Kang D. (2017). KLF2 and KLF4 control endothelial identity and vascular integrity. JCI insight 2 (4), e91700. 10.1172/jci.insight.91700 28239661 PMC5313061

[B25] Shang P. Li W. Liu G. Zhang J. Li M. Wu L. (2019a). Identification of lncRNAs and genes responsible for fatness and fatty acid composition traits between the Tibetan and Yorkshire pigs. Int. J. genomics 2019, 5070975. 10.1155/2019/5070975 31281828 PMC6589220

[B26] Shang P. Li W. Tan Z. Zhang J. Dong S. Wang K. (2020). Population genetic analysis of ten geographically isolated Tibetan pig populations. Animals open access J. MDPI 10 (8), 1297. 10.3390/ani10081297 PMC7460208 32751240

[B27] Shang P. Zhang B. Li P. Ahmed Z. Hu X. Chamba Y. (2022). Plateau adaptation gene analyses reveal transcriptomic, proteomic, and dual omics expression in the lung tissues of Tibetan and Yorkshire pigs. Animals open access J. MDPI 12 (15), 1919. 10.3390/ani12151919 PMC9367445 35953907

[B28] Shang P. Zhang B. Zhang J. Duan M. Wu L. Gong X. (2019b). Expression and single-nucleotide polymorphisms of the H-FABP gene in pigs. Gene 710, 156–160. 10.1016/j.gene.2019.05.061 31173805

[B29] Shao F. Zhang R. Don L. Ying K. (2011). Overexpression of gelsolin-like actin-capping protein is associated with progression of lung adenocarcinoma. Tohoku J. Exp. Med. 225 (2), 95–101. 10.1620/tjem.225.95 21908955

[B30] Shao X. Dong X. Cai J. Tang C. Xie K. Yan Z. (2020). Oxygen enrichment ameliorates cardiorespiratory alterations induced by chronic high-altitude hypoxia in rats. Front. physiology 11, 616145. 10.3389/fphys.2020.616145 PMC7817980 33488404

[B31] Silacci P. Mazzolai L. Gauci C. Stergiopulos N. Yin H. Hayoz D. (2004). Gelsolin superfamily proteins: key regulators of cellular functions. Cell. Mol. life Sci. CMLS 61, 2614–2623. 10.1007/s00018-004-4225-6 15526166 PMC11924436

[B32] Storz J. Scott G. Cheviron Z. (2010). Phenotypic plasticity and genetic adaptation to high-altitude hypoxia in vertebrates. J. Exp. Biol. 213, 4125–4136. 10.1242/jeb.048181 21112992 PMC2992463

[B33] Sun H. Kwiatkowska K. Wooten D. Yin H. (1995). Effects of CapG overexpression on agonist-induced motility and second messenger generation. J. Cell Biol. 129 (1), 147–156. 10.1083/jcb.129.1.147 7698981 PMC2120377

[B34] Wang J. Xiao J. Liu X. Gao Y. Luo Z. Gu X. (2021). Tandem mass tag-labeled quantitative proteomic analysis of tenderloins between Tibetan and Yorkshire pigs. Meat Sci. 172, 108343. 10.1016/j.meatsci.2020.108343 33099162

[B35] Wang Y. Wang L. Yu W. Gao D. You G. Li P. (2017). A PEGylated bovine hemoglobin as a potent hemoglobin-based oxygen carrier. Biotechnol. Prog. 33 (1), 252–260. 10.1002/btpr.2380 27696787

[B36] Xu X. Hu H. Wang X. Ye W. Su H. Hu Y. (2016). Involvement of CapG in proliferation and apoptosis of pulmonary arterial smooth muscle cells and in hypoxia-induced pulmonary hypertension rat model. Exp. lung Res. 42 (3), 142–153. 10.3109/01902148.2016.1160304 27093378

[B37] Yi X. Liang Y. Huerta-Sanchez E. Jin X. Cuo Z. Pool J. (2010). Sequencing of 50 human exomes reveals adaptation to high altitude. Sci. (New York, N.Y.) 329 (5987), 75–78. 10.1126/science.1190371 PMC3711608 20595611

[B38] Yuan H. Liu X. Wang Z. Ren Y. Li Y. Gao C. (2022). Alternative splicing signature of alveolar type II epithelial cells of Tibetan pigs under hypoxia-induced. Front. veterinary Sci. 9, 984703. 10.3389/fvets.2022.984703 PMC9523697 36187824

[B39] Zhang B. Chamba Y. Shang P. Wang Z. Ma J. Wang L. (2017). Comparative transcriptomic and proteomic analyses provide insights into the key genes involved in high-altitude adaptation in the Tibetan pig. Sci. Rep. 7 (1), 3654. 10.1038/s41598-017-03976-3 28623314 PMC5473931

[B40] Zhang J. Chen G. (2009). Hypoxia-HIF-1alpha-C/EBPalpha/Runx1 signaling in leukemic cell differentiation. Pathophysiol. official J. Int. Soc. Pathophysiol. 16 (4), 297–303. 10.1016/j.pathophys.2009.02.005 19285840

[B41] Zhang R. Zhou L. Li Q. Liu J. Yao W. Wan H. (2009). Up-regulation of two actin-associated proteins prompts pulmonary artery smooth muscle cell migration under hypoxia. Am. J. Respir. Cell Mol. Biol. 41 (4), 467–475. 10.1165/rcmb.2008-0333OC 19188659

[B42] Zhang Y. Hu Y. Wang X. Jiang Q. Zhao H. Wang J. (2019). Population structure, and selection signatures underlying high-altitude adaptation inferred from genome-wide copy number variations in Chinese indigenous cattle. Front. Genet. 10, 1404. 10.3389/fgene.2019.01404 32117428 PMC7033542

