# Supplementary figures and images for: Exploring the role of the CapG gene in hypoxia adaptation in Tibetan pigs
Source: Front Genet. 2024 Apr 12;15:1339683. doi: 10.3389/fgene.2024.1339683 (PMC11045884; doi:10.3389/fgene.2024.1339683)

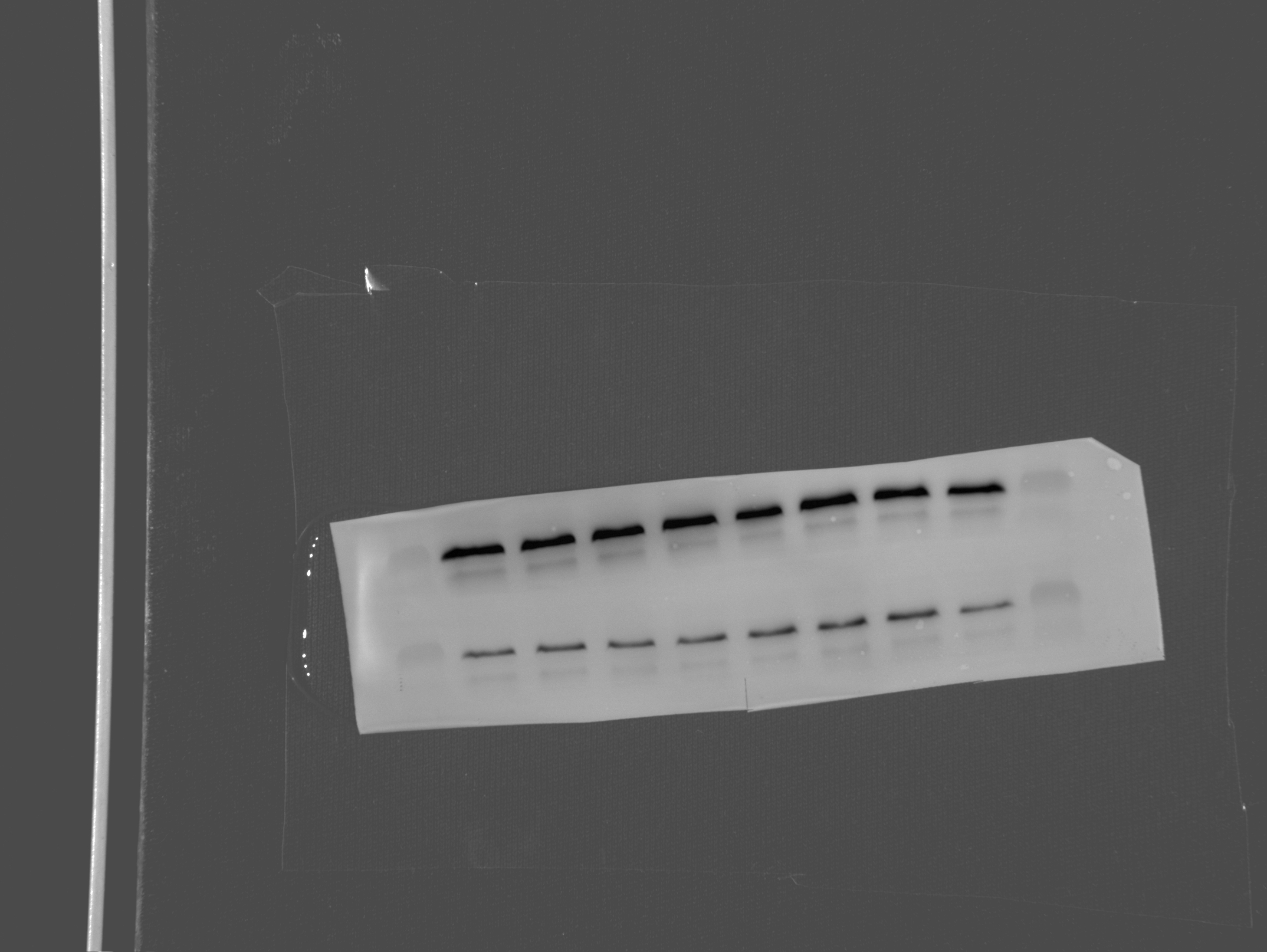

Supplement: Supplementary file 2 [file DataSheet4.ZIP › 4.Protein_data/Figure3/Reference protein.jpg]

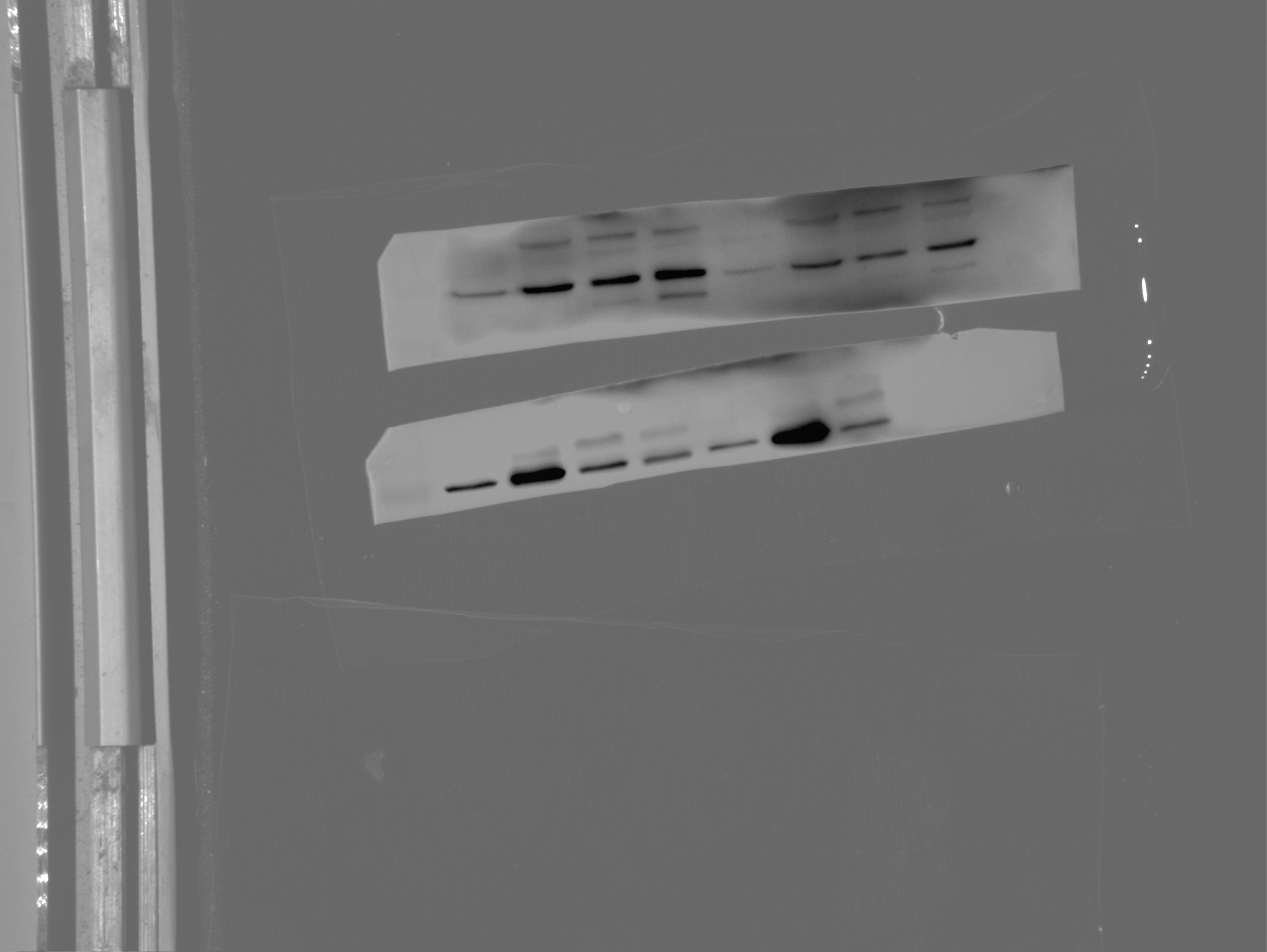

Supplement: Supplementary file 2 [file DataSheet4.ZIP › 4.Protein_data/Figure3/Target protein.jpg]

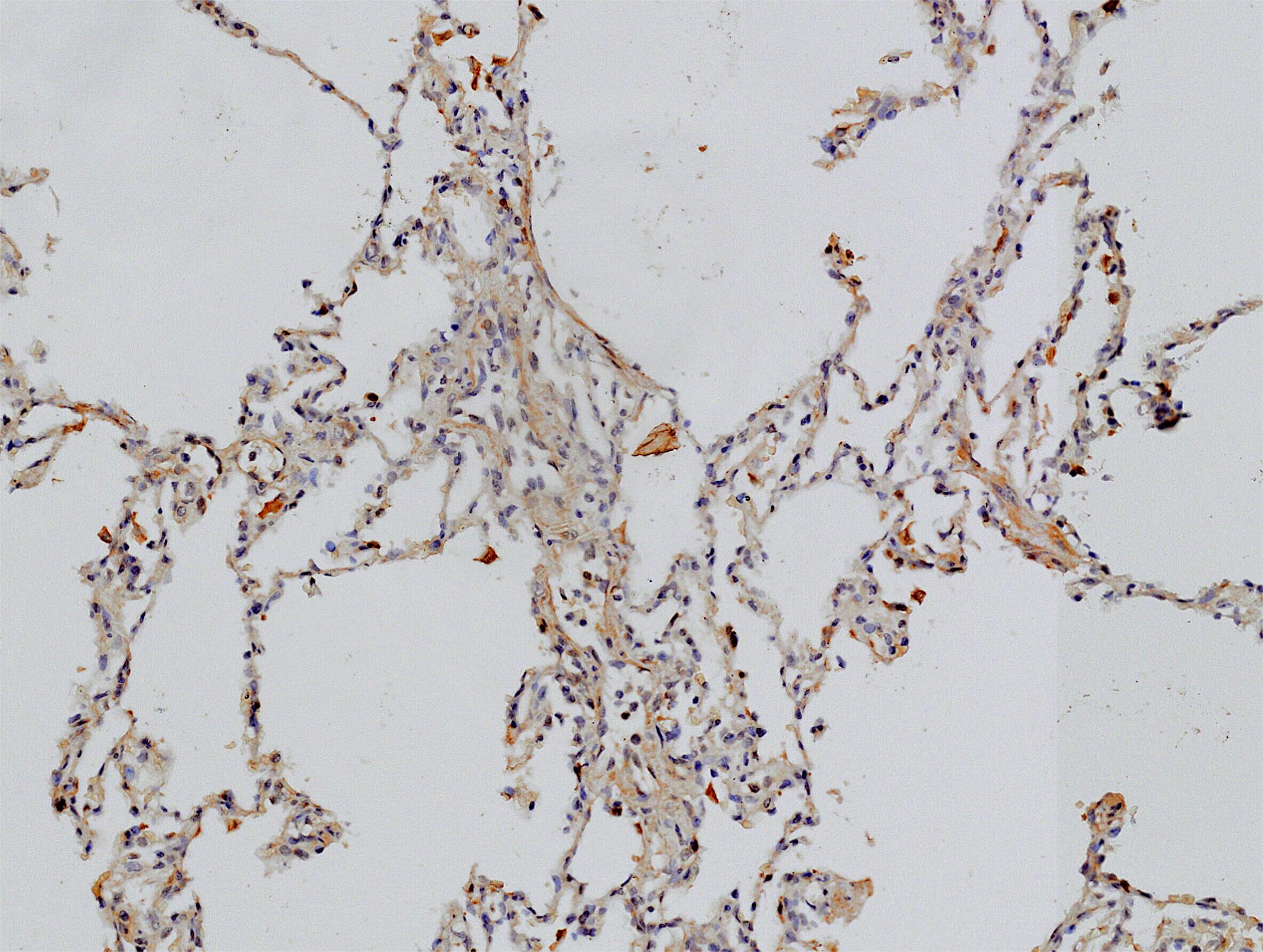

Supplement: Supplementary file 4 [file DataSheet6.ZIP › TP_IHC_Original image/T1/图像_01.jpg]

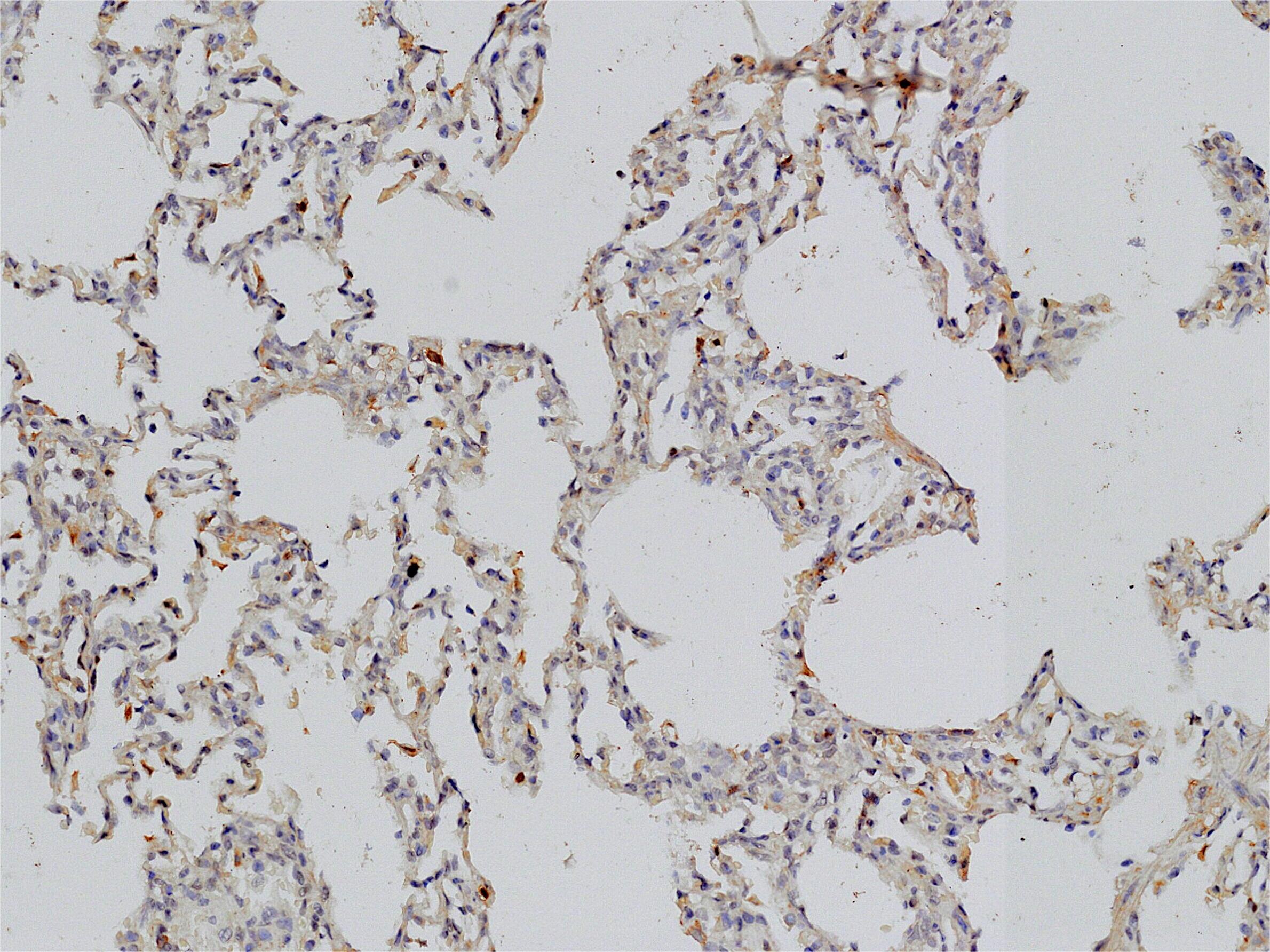

Supplement: Supplementary file 4 [file DataSheet6.ZIP › TP_IHC_Original image/T1/图像_02.jpg]

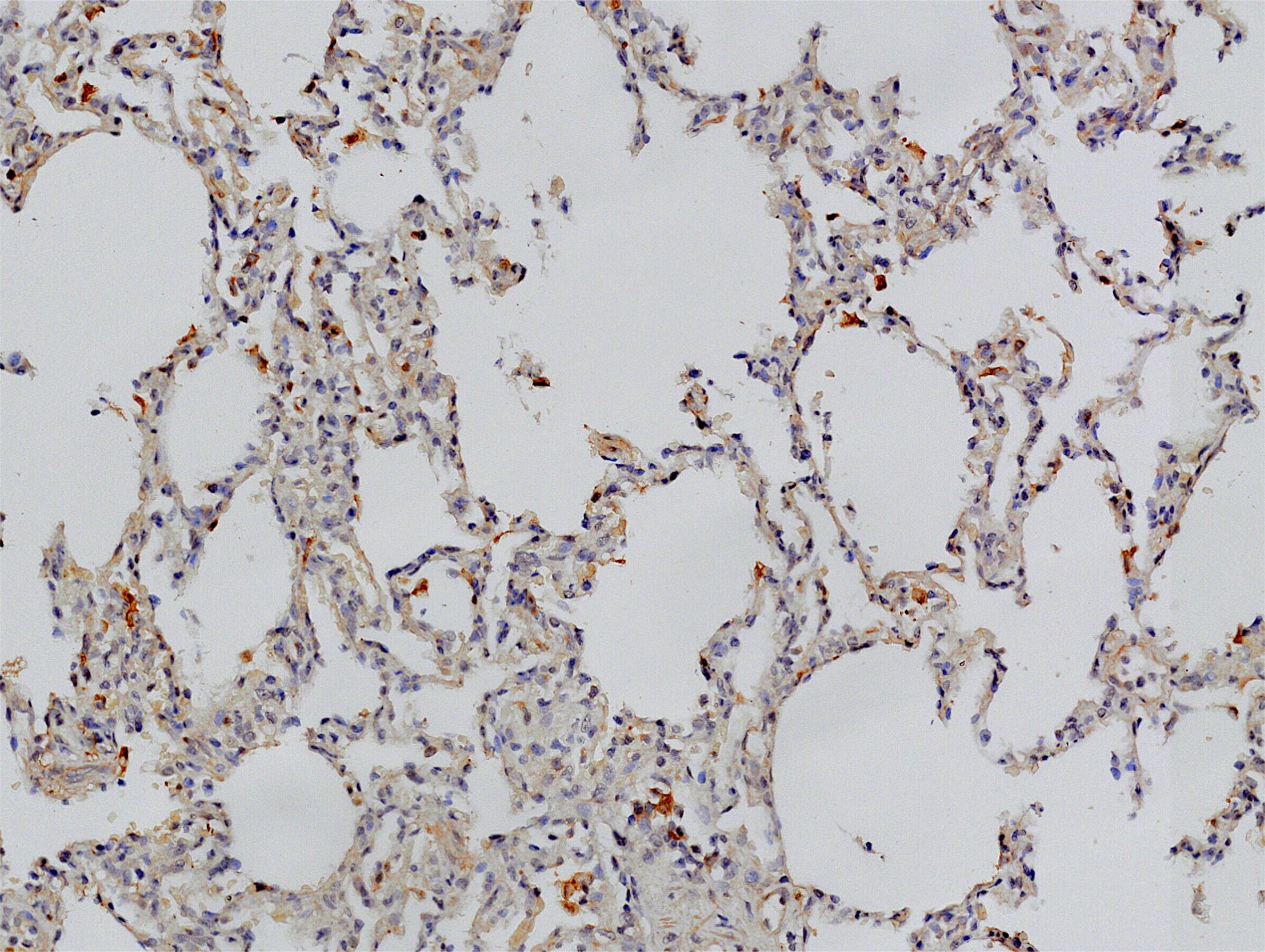

Supplement: Supplementary file 4 [file DataSheet6.ZIP › TP_IHC_Original image/T1/图像_03.jpg]

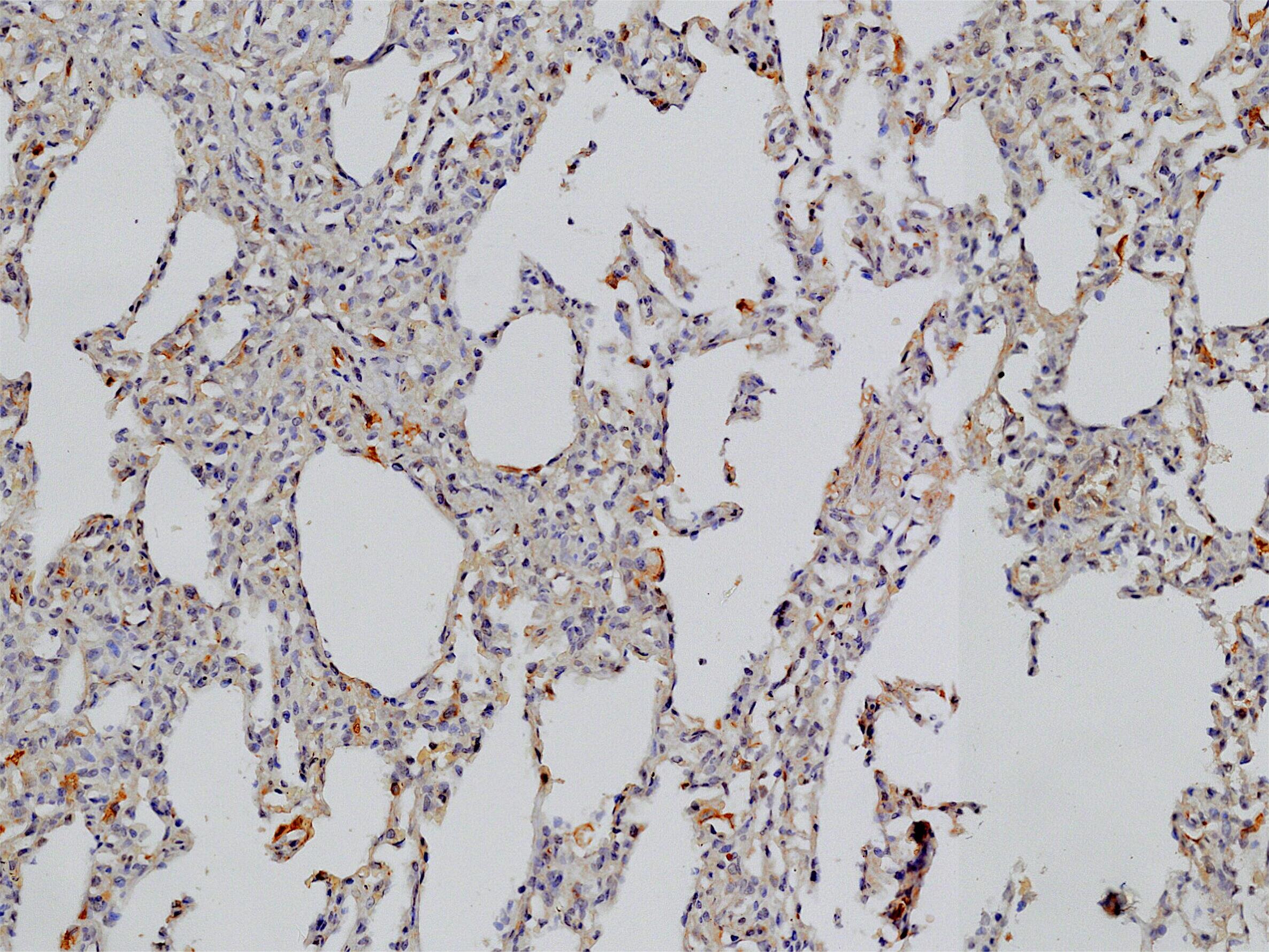

Supplement: Supplementary file 4 [file DataSheet6.ZIP › TP_IHC_Original image/T1/图像_04.jpg]

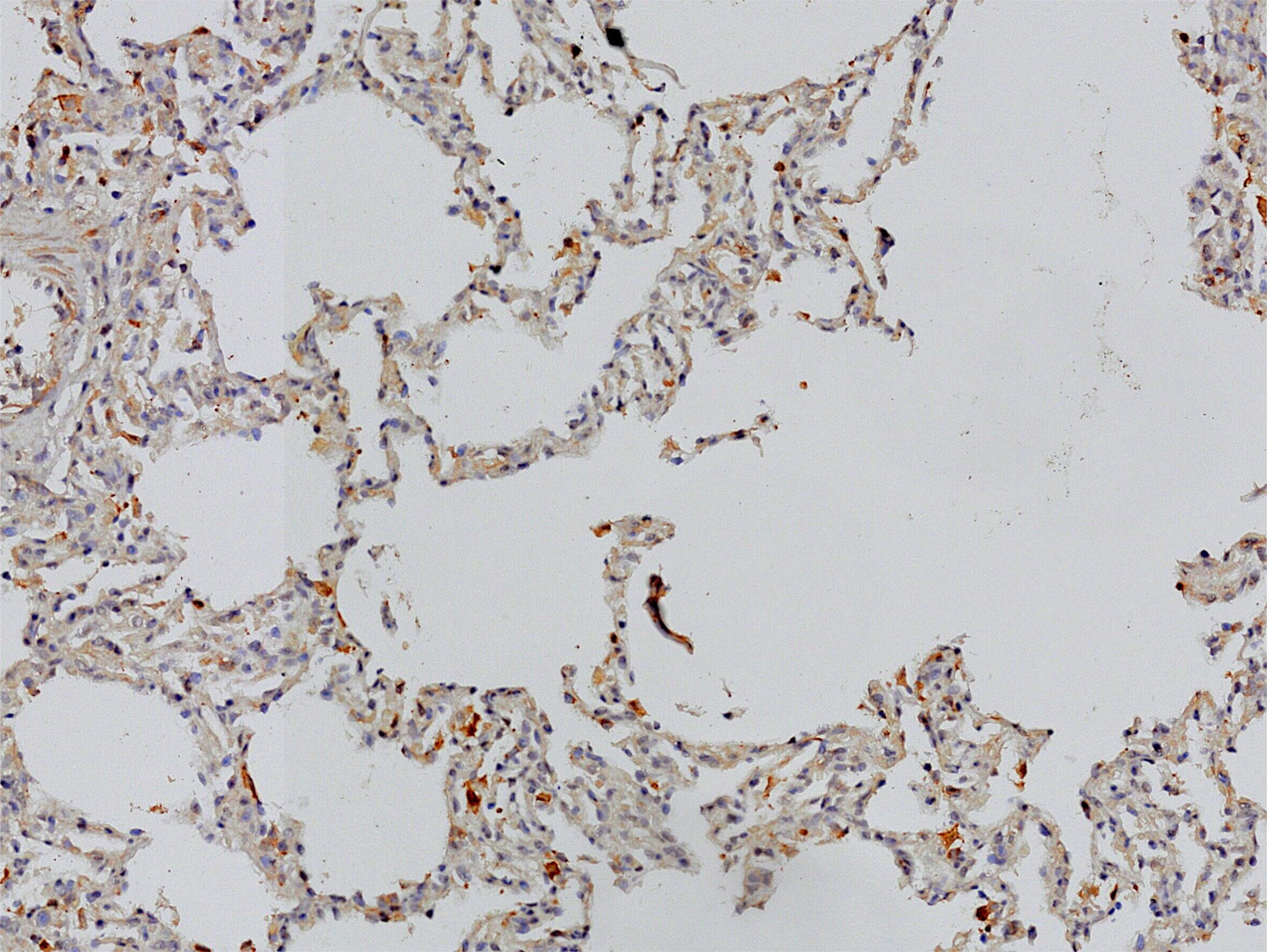

Supplement: Supplementary file 4 [file DataSheet6.ZIP › TP_IHC_Original image/T1/图像_05.jpg]

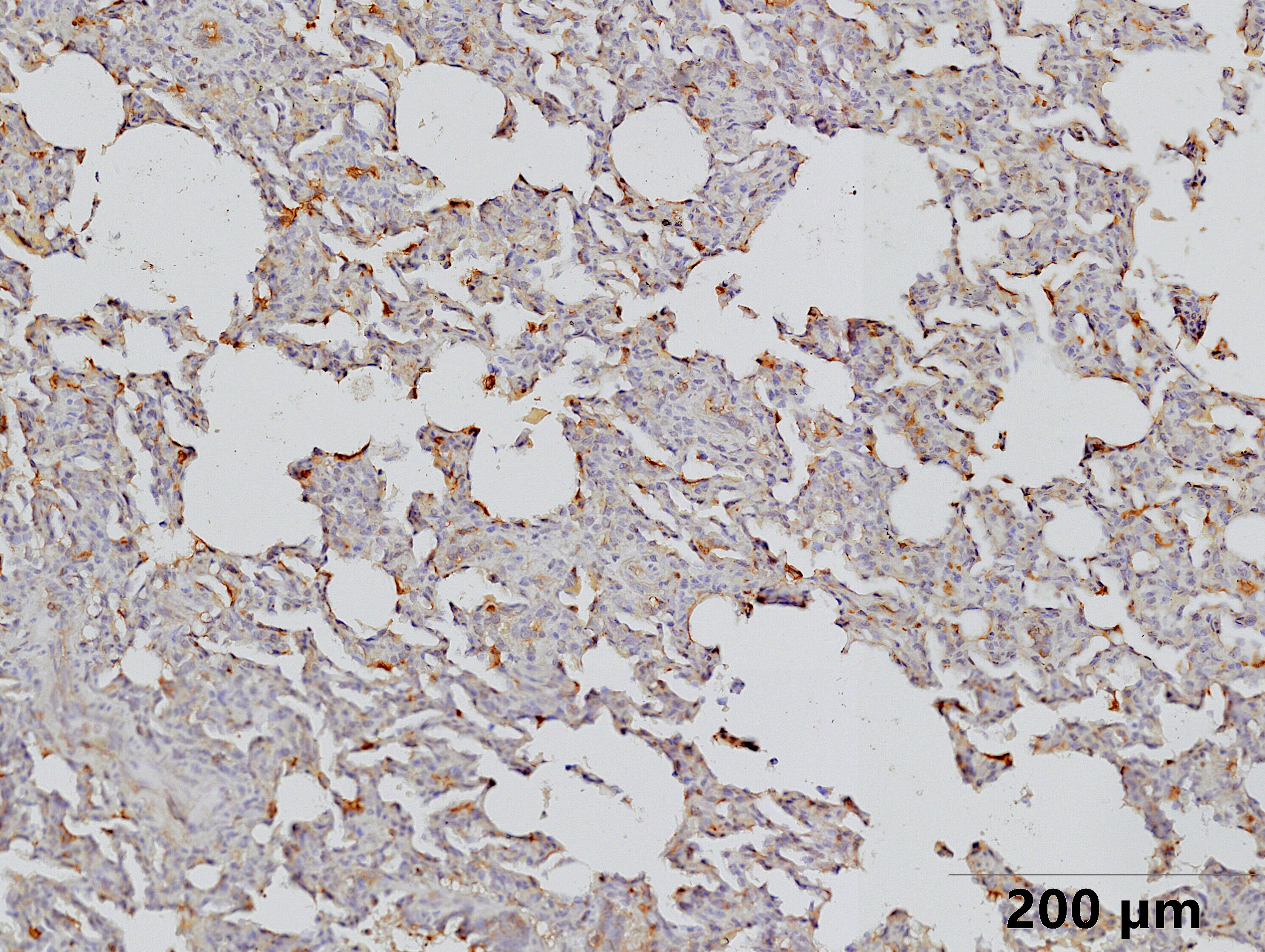

Supplement: Supplementary file 4 [file DataSheet6.ZIP › TP_IHC_Original image/T2/图像_01.jpg]

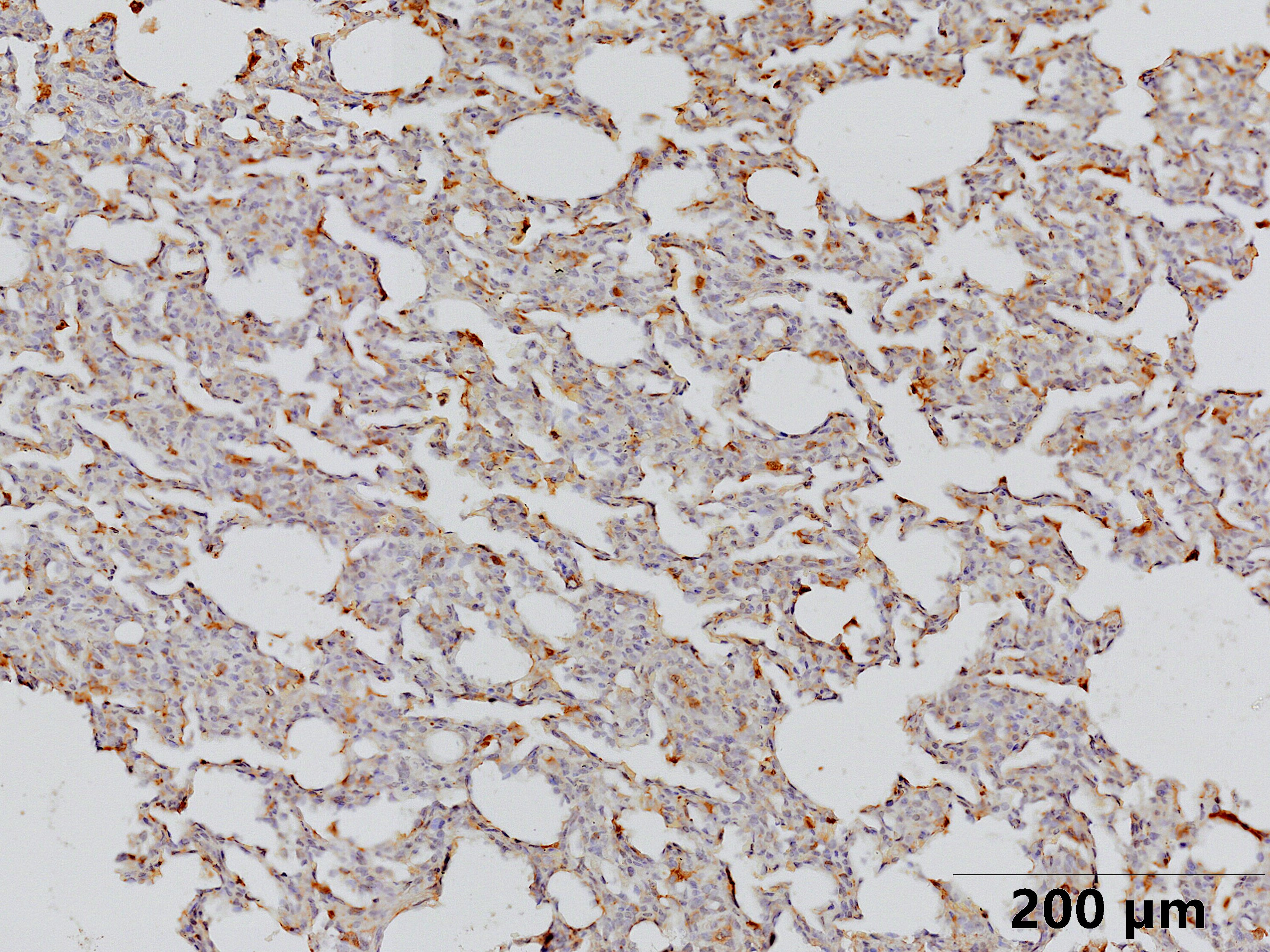

Supplement: Supplementary file 4 [file DataSheet6.ZIP › TP_IHC_Original image/T2/图像_02.jpg]

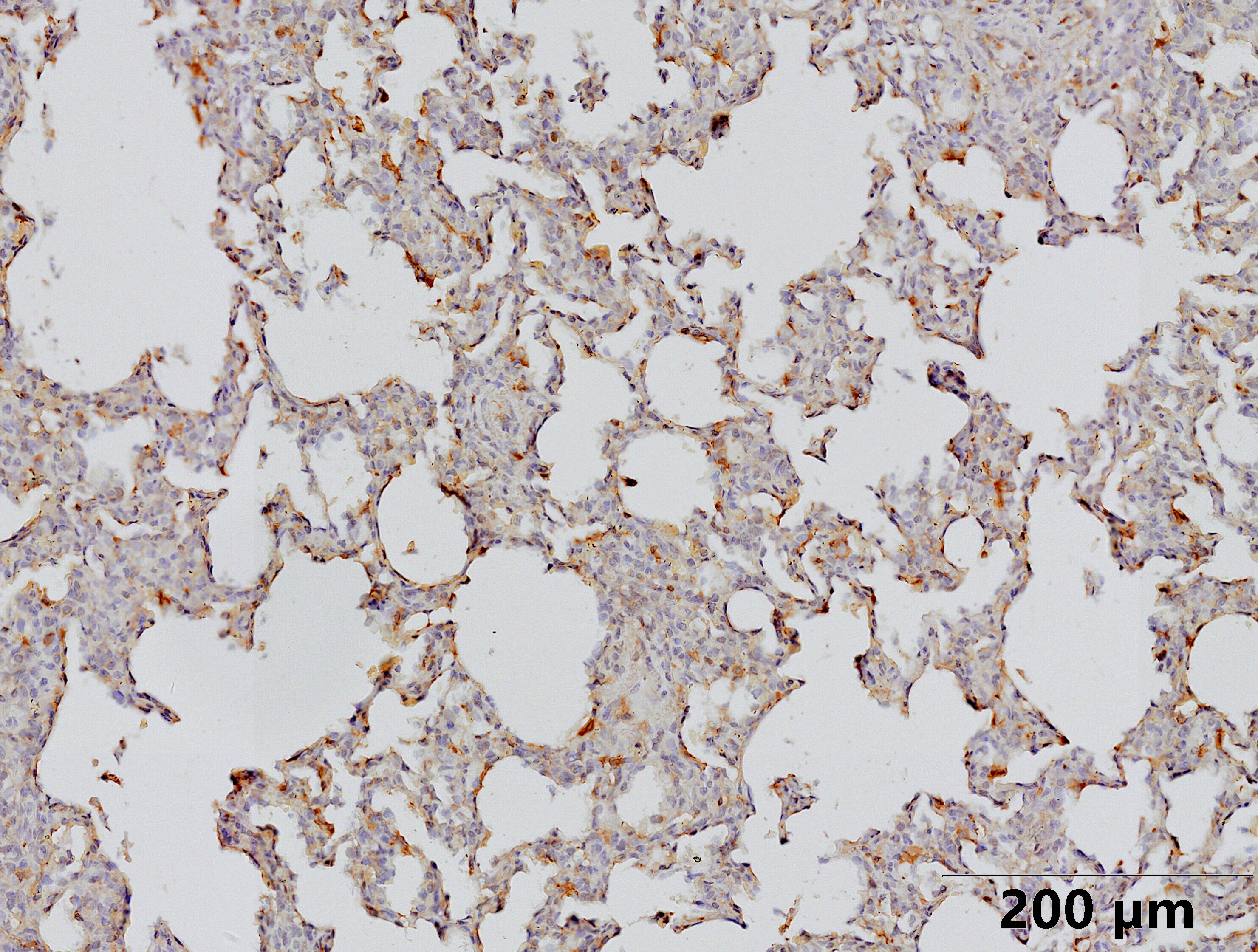

Supplement: Supplementary file 4 [file DataSheet6.ZIP › TP_IHC_Original image/T2/图像_03.jpg]

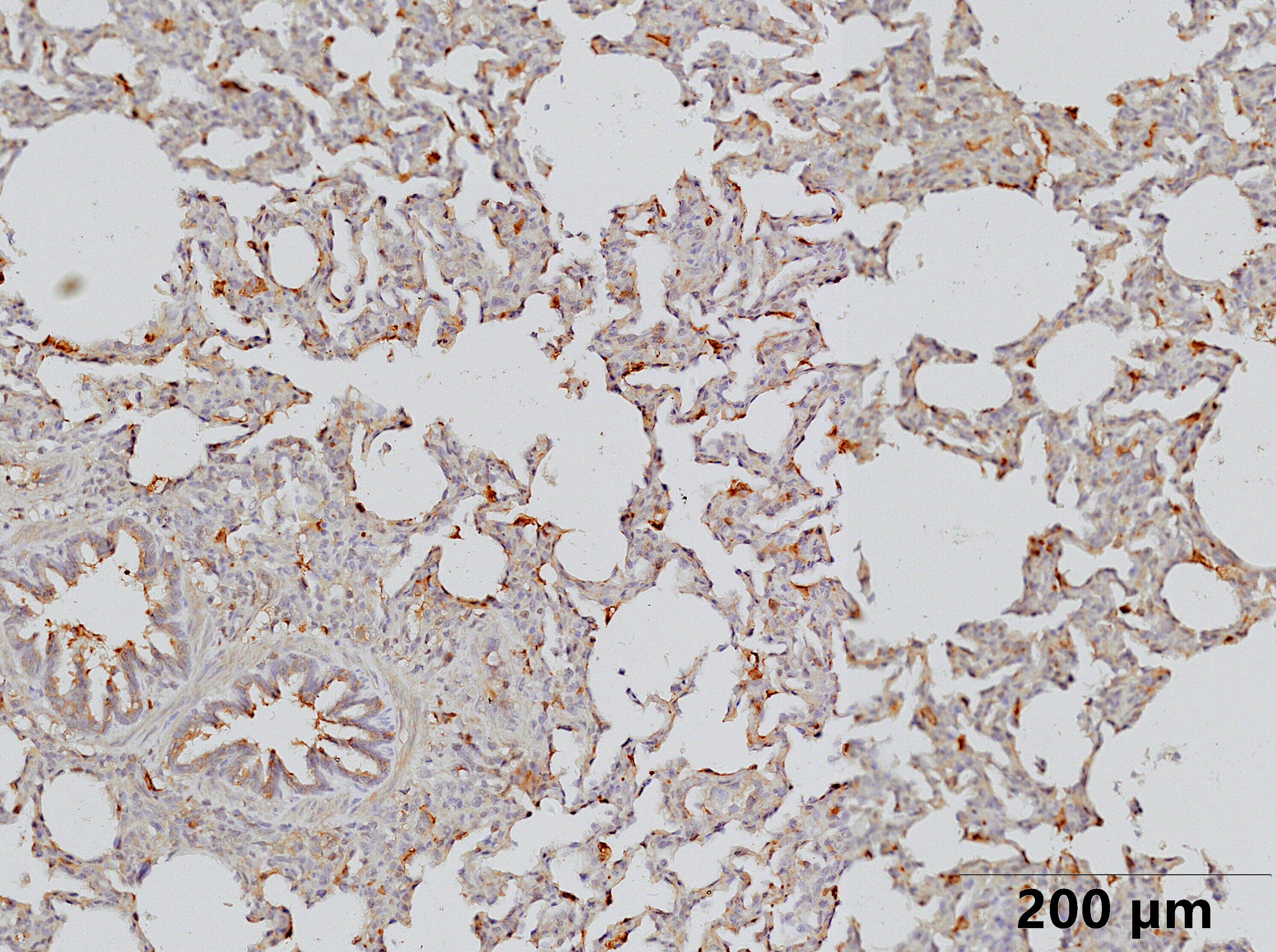

Supplement: Supplementary file 4 [file DataSheet6.ZIP › TP_IHC_Original image/T2/图像_04.jpg]

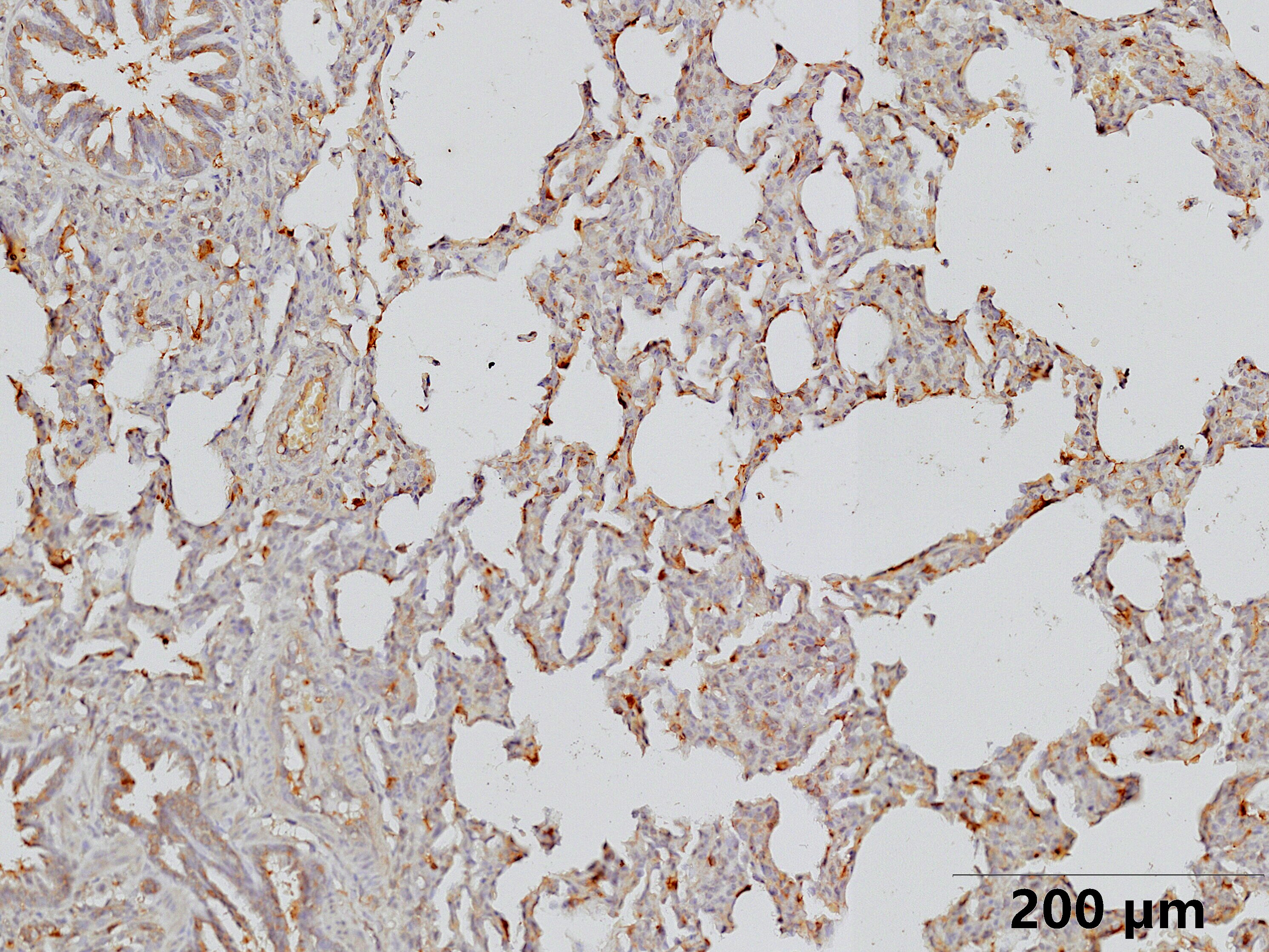

Supplement: Supplementary file 4 [file DataSheet6.ZIP › TP_IHC_Original image/T2/图像_05.jpg]

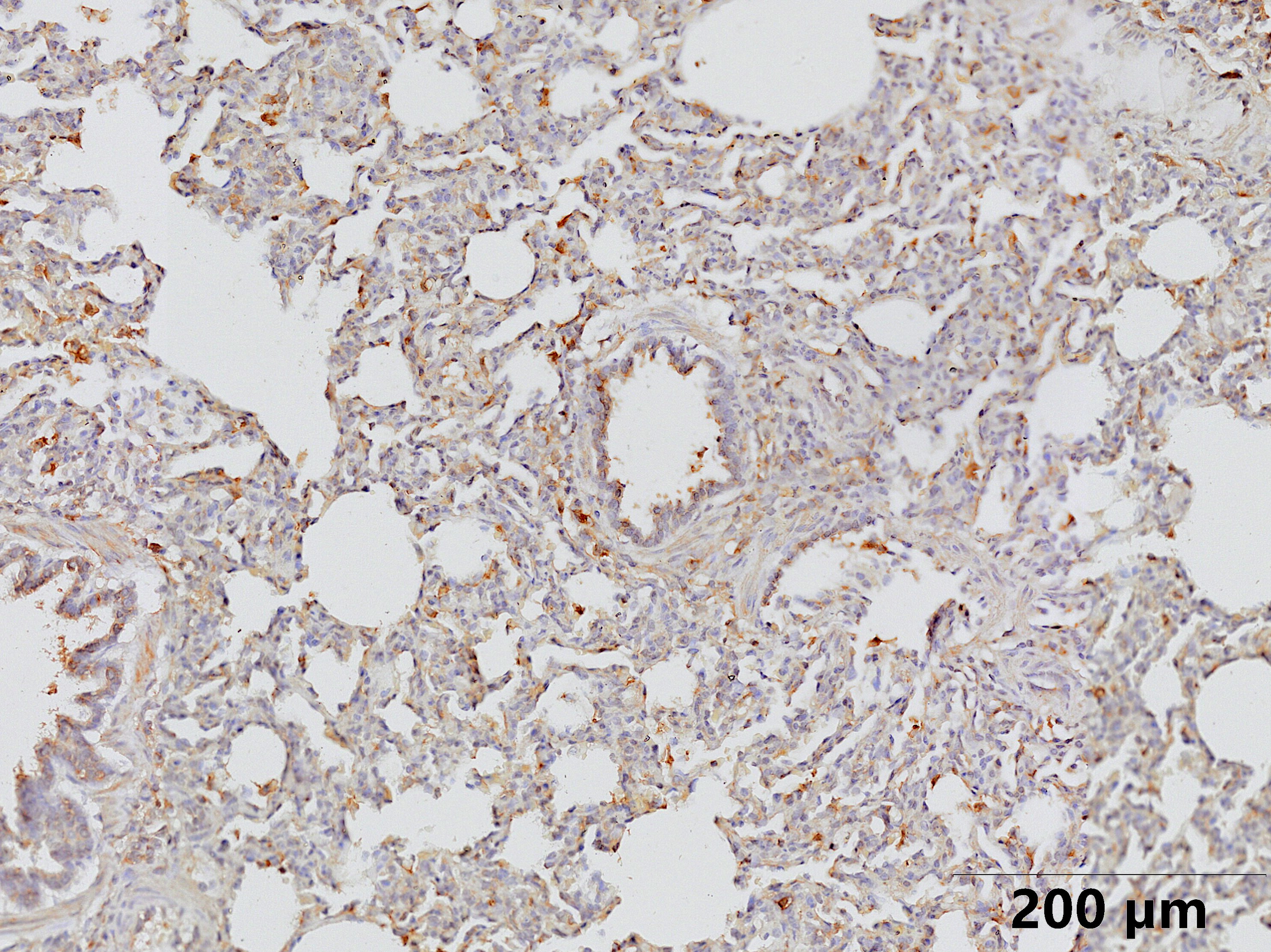

Supplement: Supplementary file 4 [file DataSheet6.ZIP › TP_IHC_Original image/T3/图像_01.jpg]

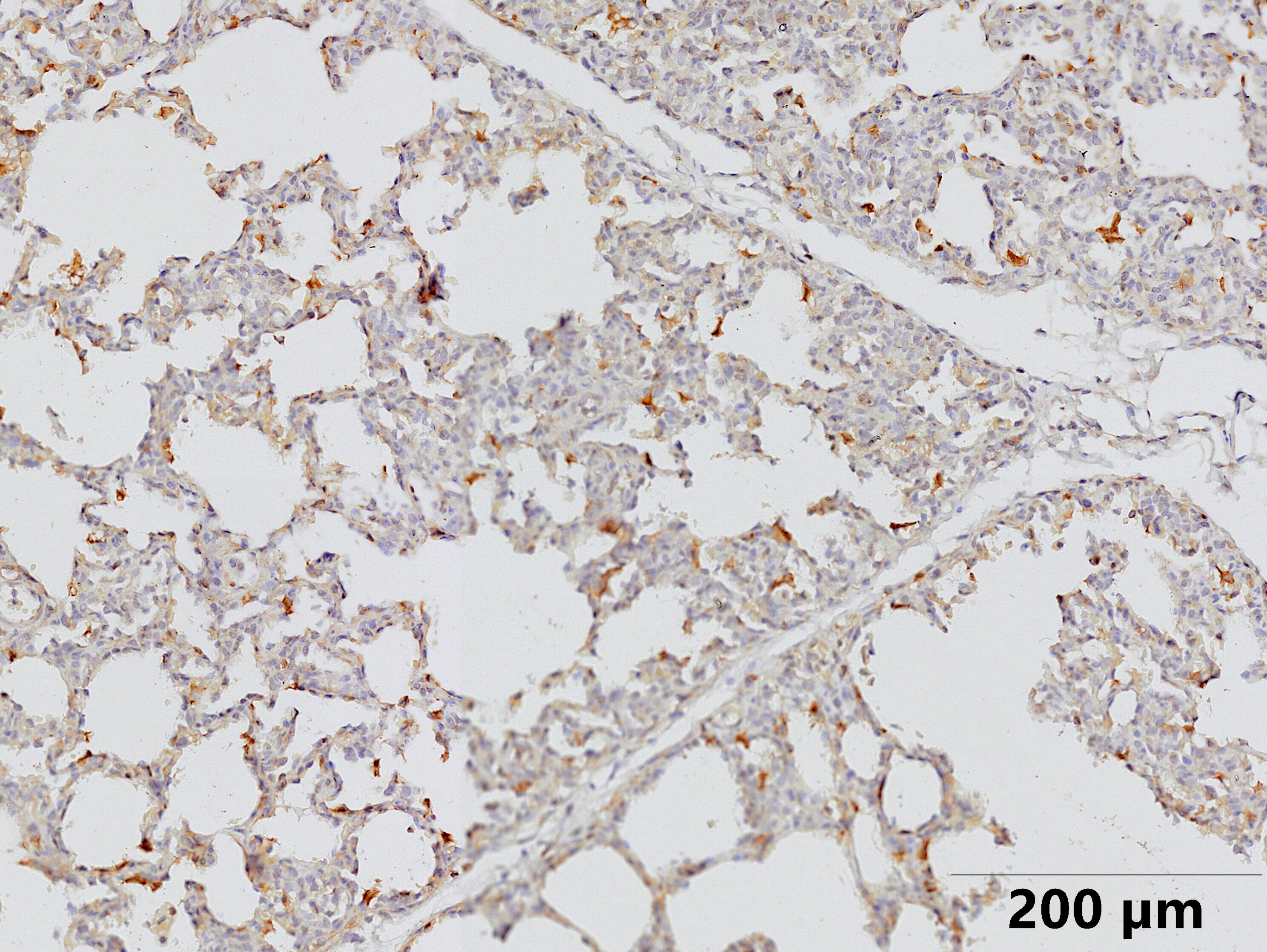

Supplement: Supplementary file 4 [file DataSheet6.ZIP › TP_IHC_Original image/T3/图像_02.jpg]

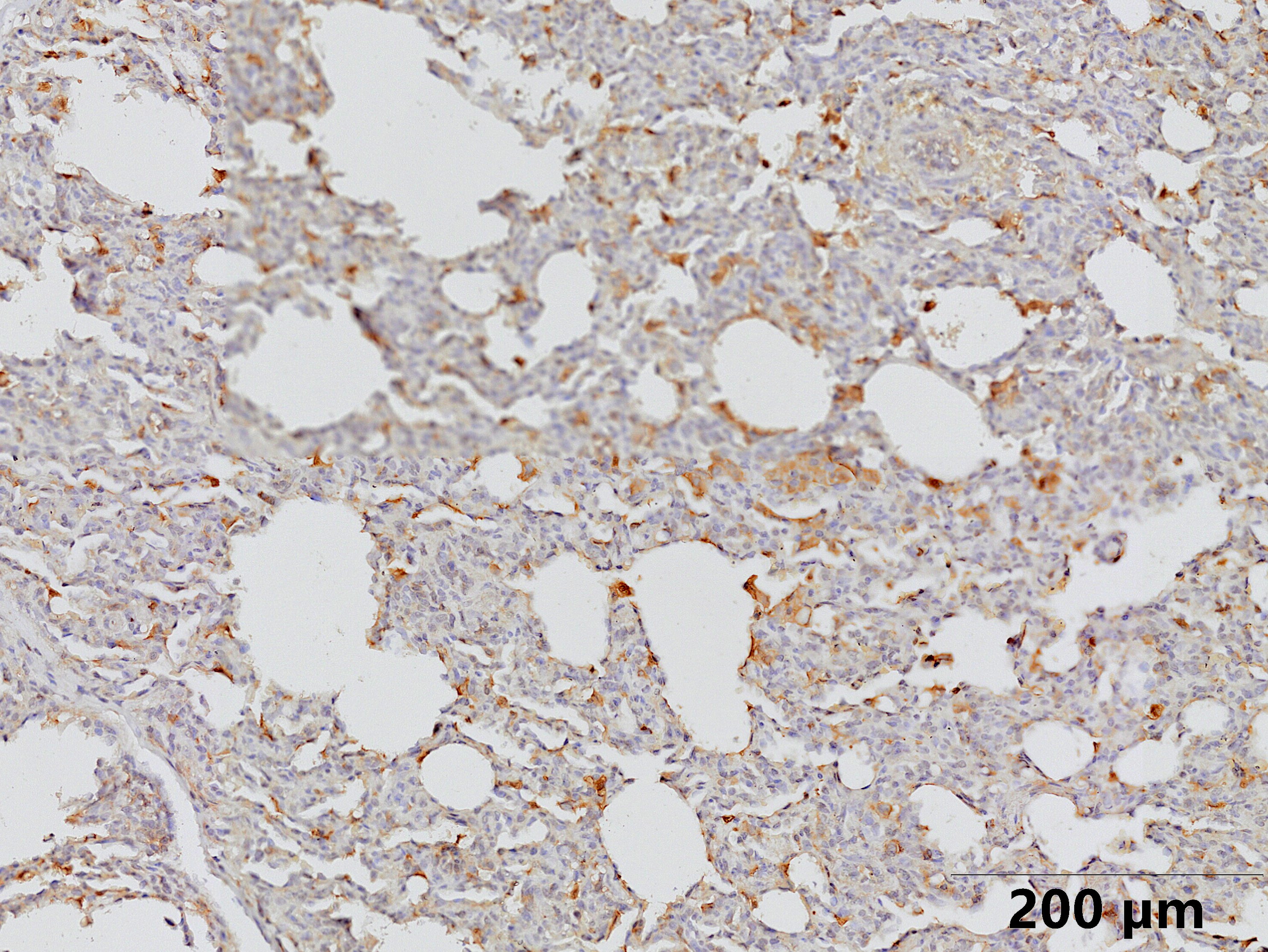

Supplement: Supplementary file 4 [file DataSheet6.ZIP › TP_IHC_Original image/T3/图像_03.jpg]

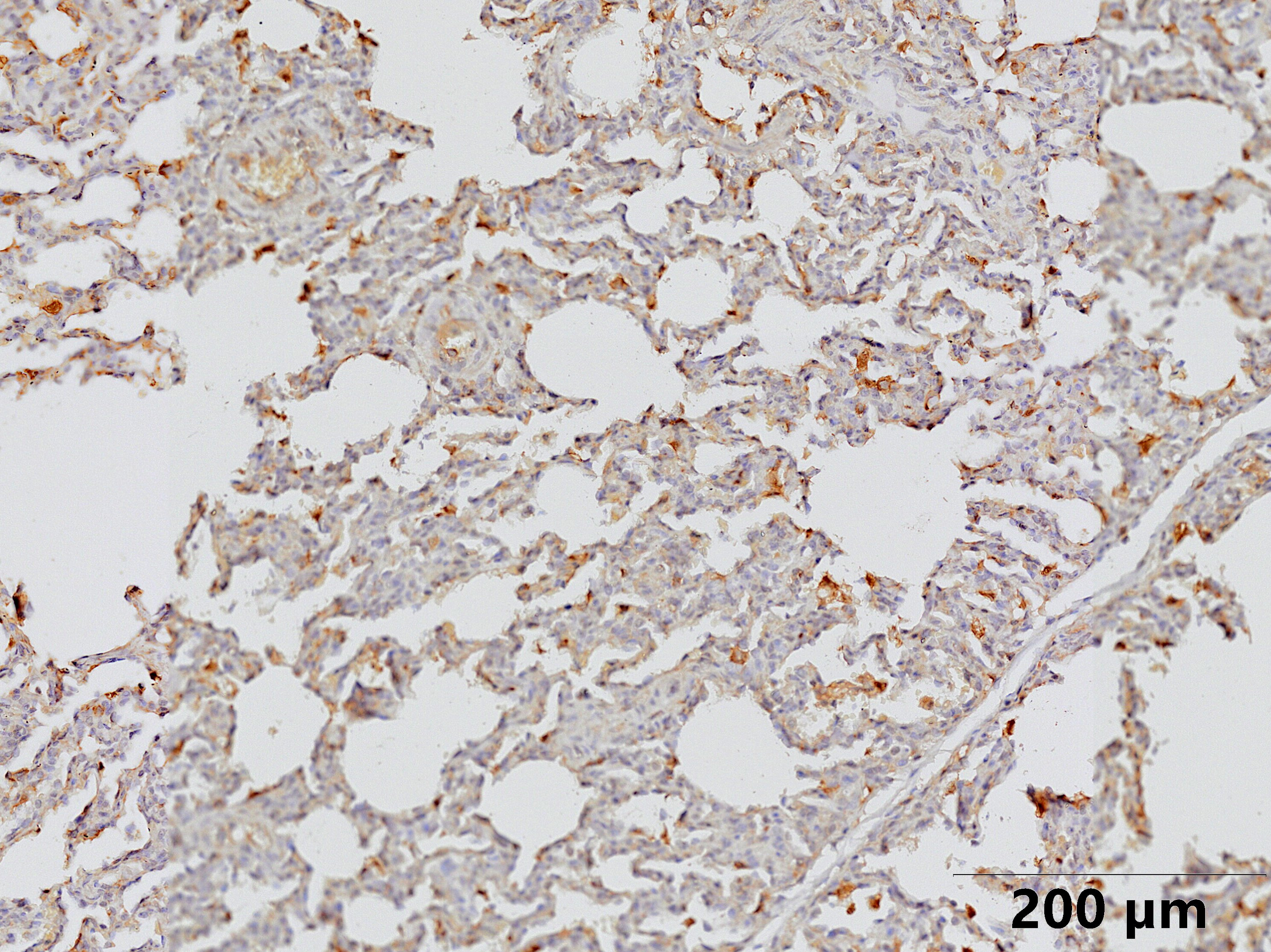

Supplement: Supplementary file 4 [file DataSheet6.ZIP › TP_IHC_Original image/T3/图像_04.jpg]

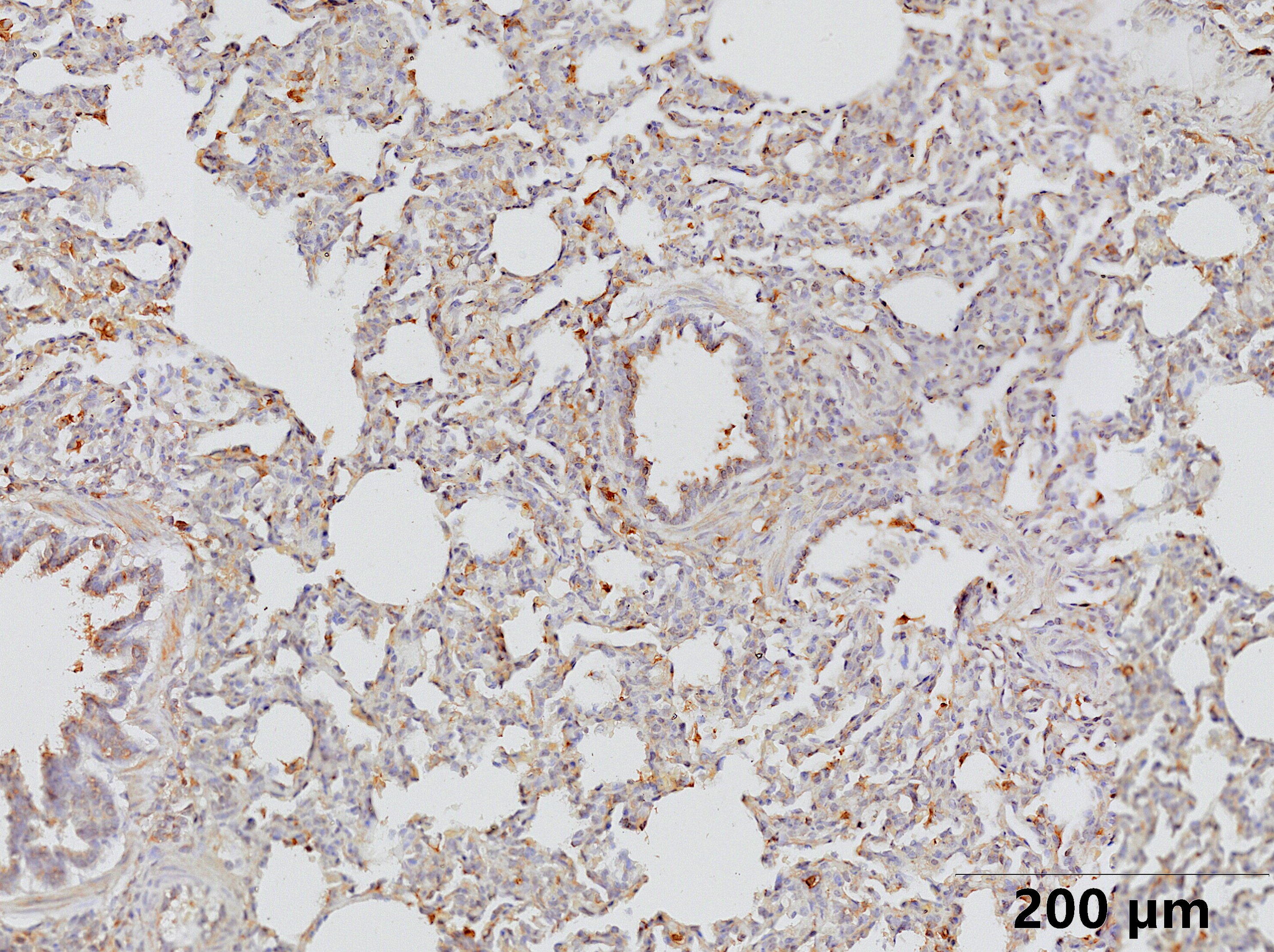

Supplement: Supplementary file 4 [file DataSheet6.ZIP › TP_IHC_Original image/T3/图像_05.jpg]

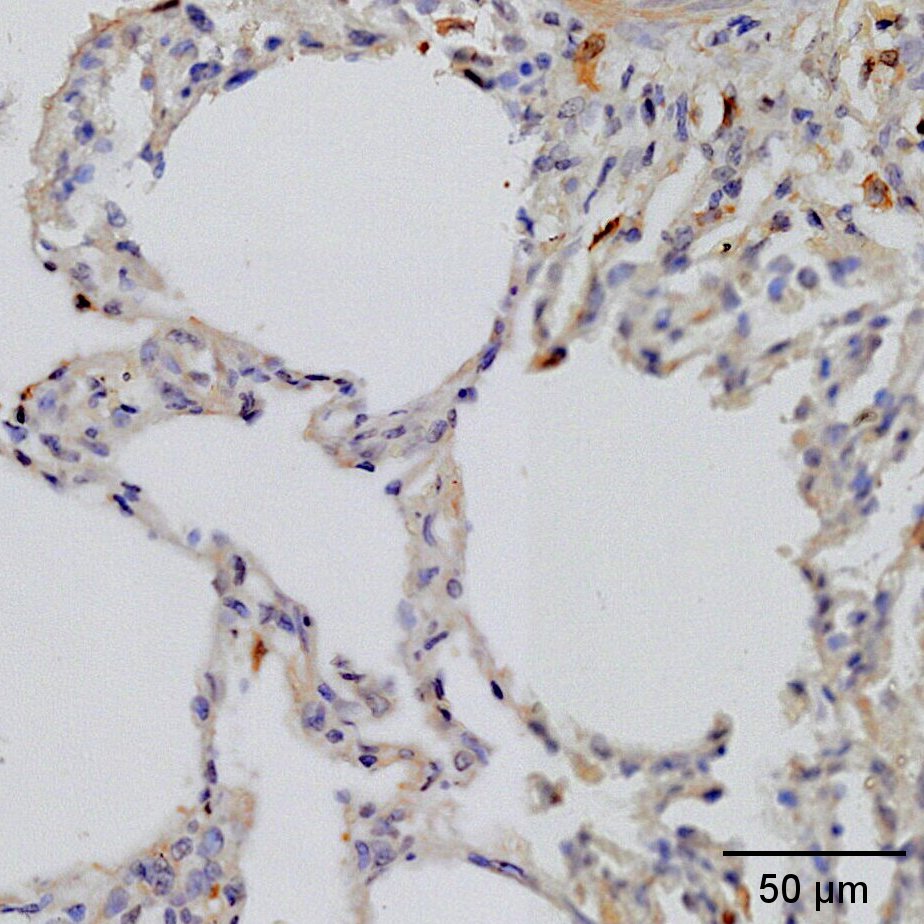

Supplement: Supplementary file 6 [file DataSheet5.ZIP › 5.IHC_data/Figure4/TP.jpg]

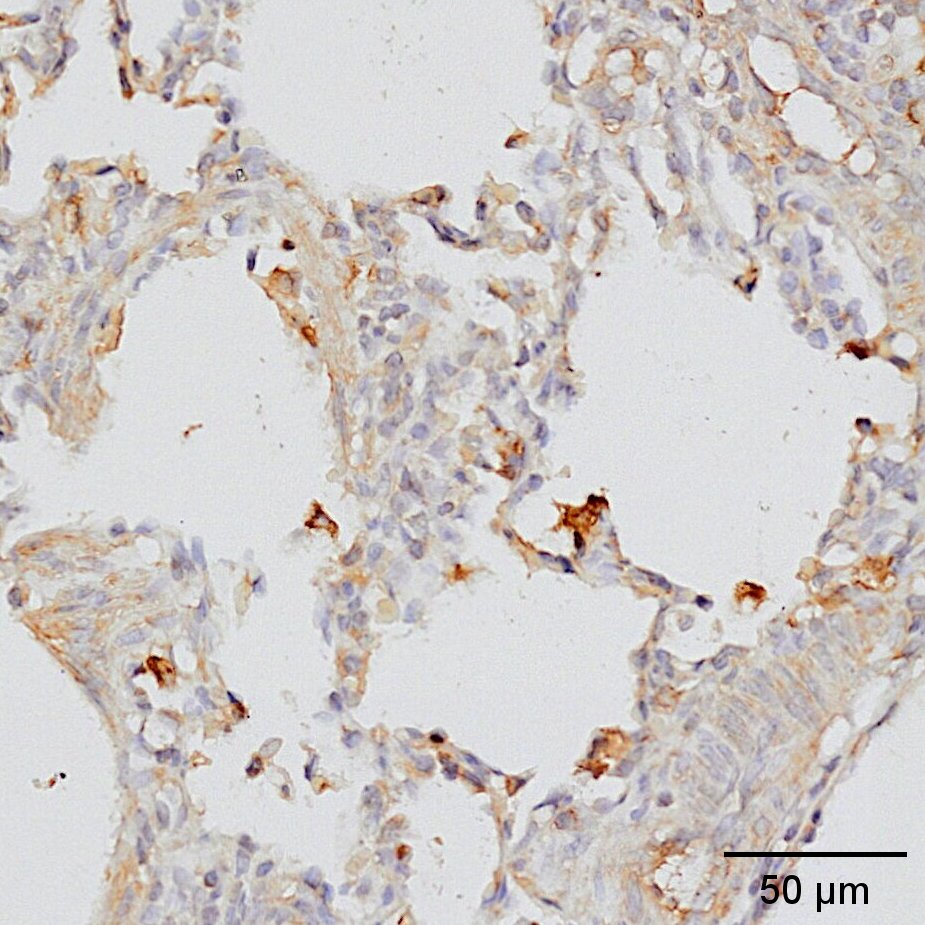

Supplement: Supplementary file 6 [file DataSheet5.ZIP › 5.IHC_data/Figure4/YY.jpg]

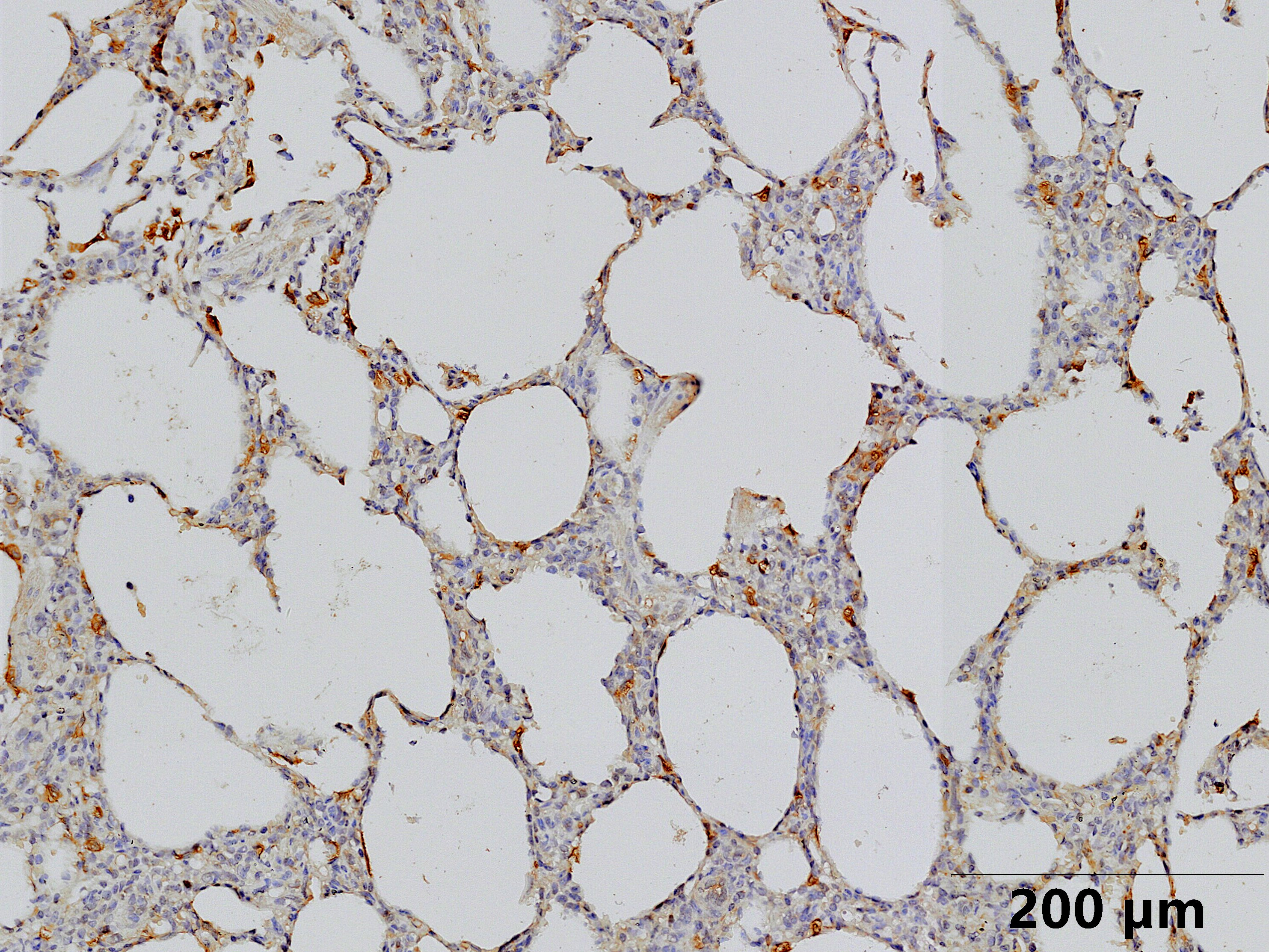

Supplement: Supplementary file 7 [file DataSheet7.ZIP › YY_IHC_Original image/Y1/图像_01.jpg]

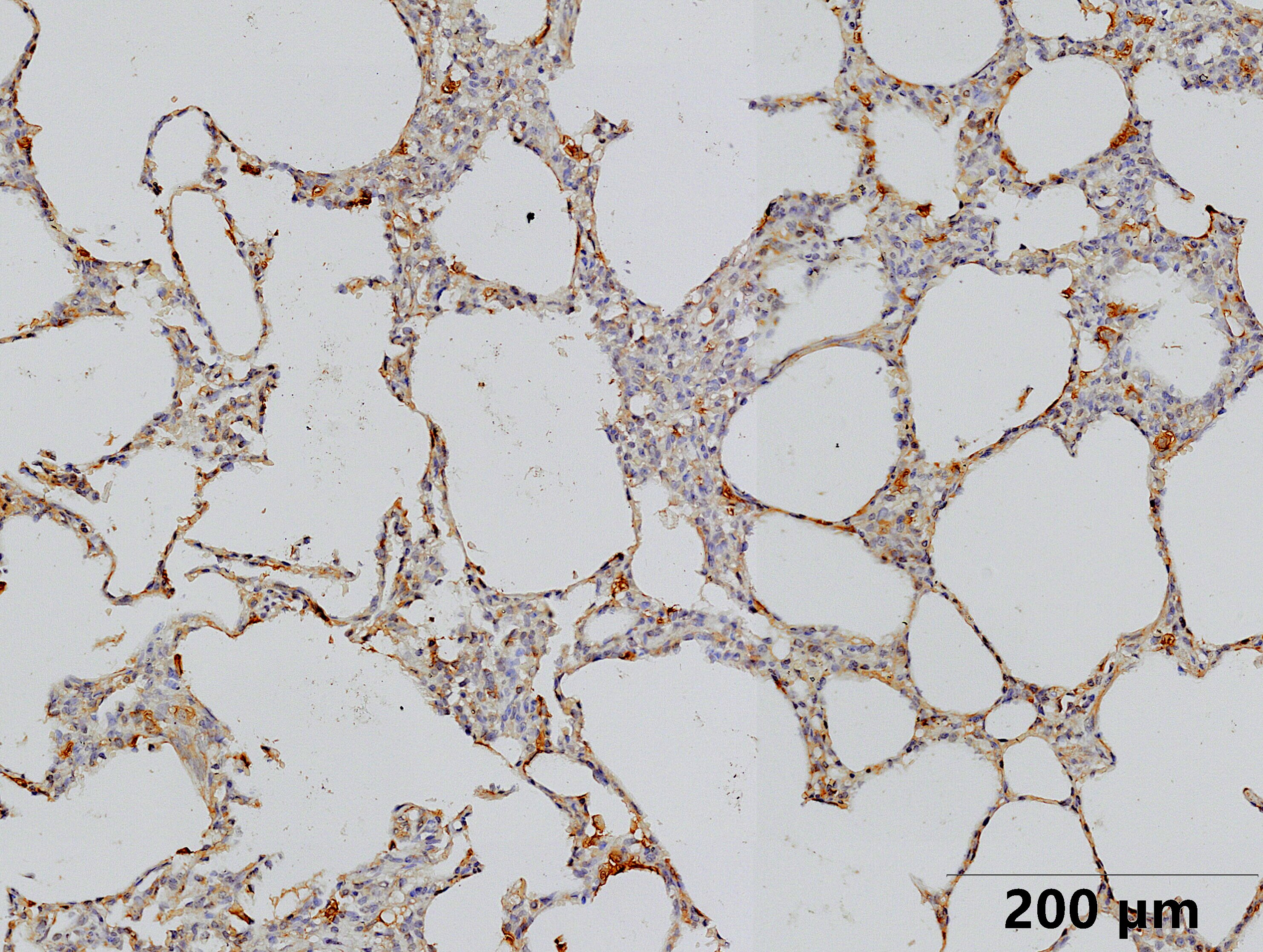

Supplement: Supplementary file 7 [file DataSheet7.ZIP › YY_IHC_Original image/Y1/图像_02.jpg]

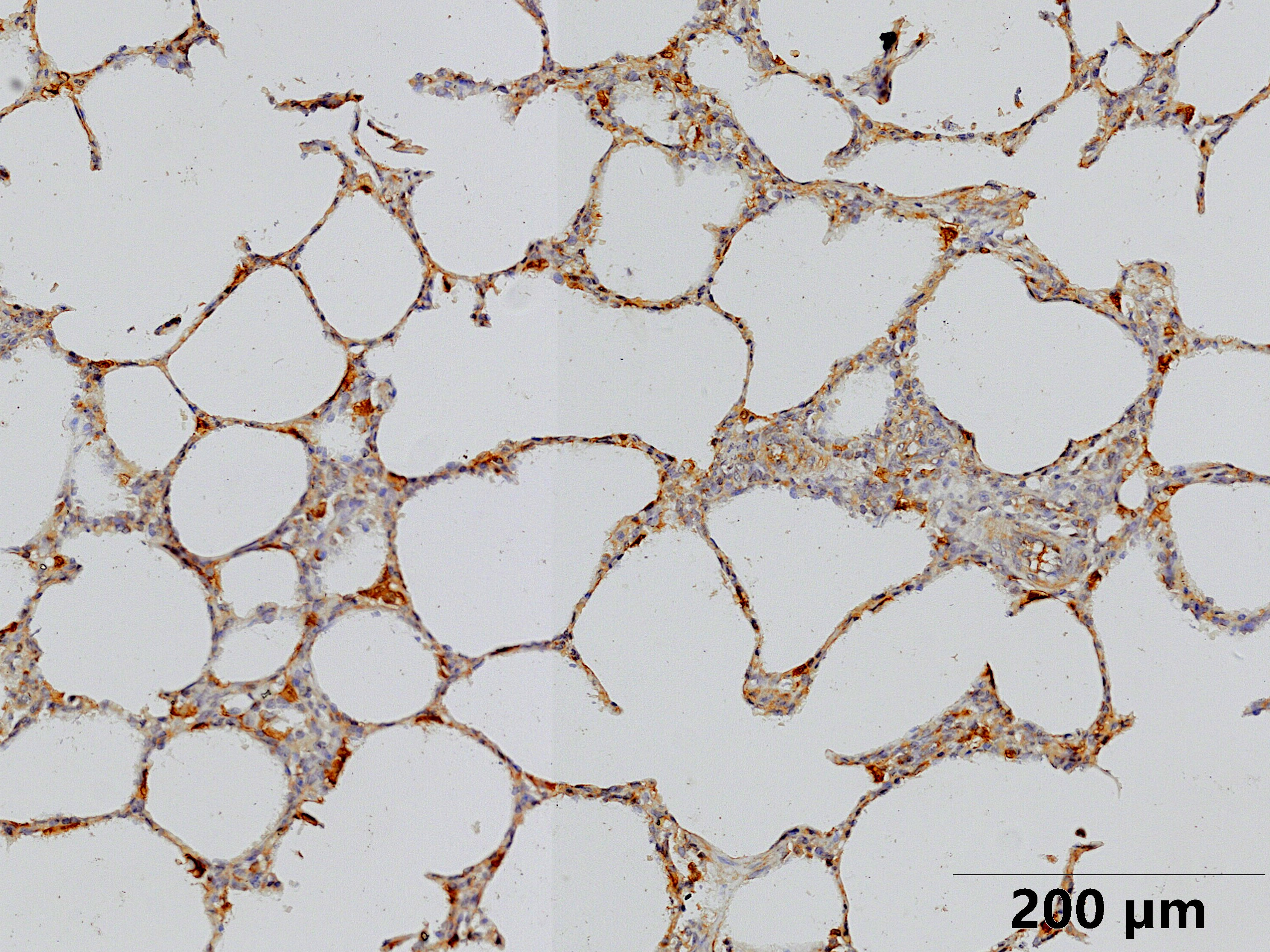

Supplement: Supplementary file 7 [file DataSheet7.ZIP › YY_IHC_Original image/Y1/图像_03.jpg]

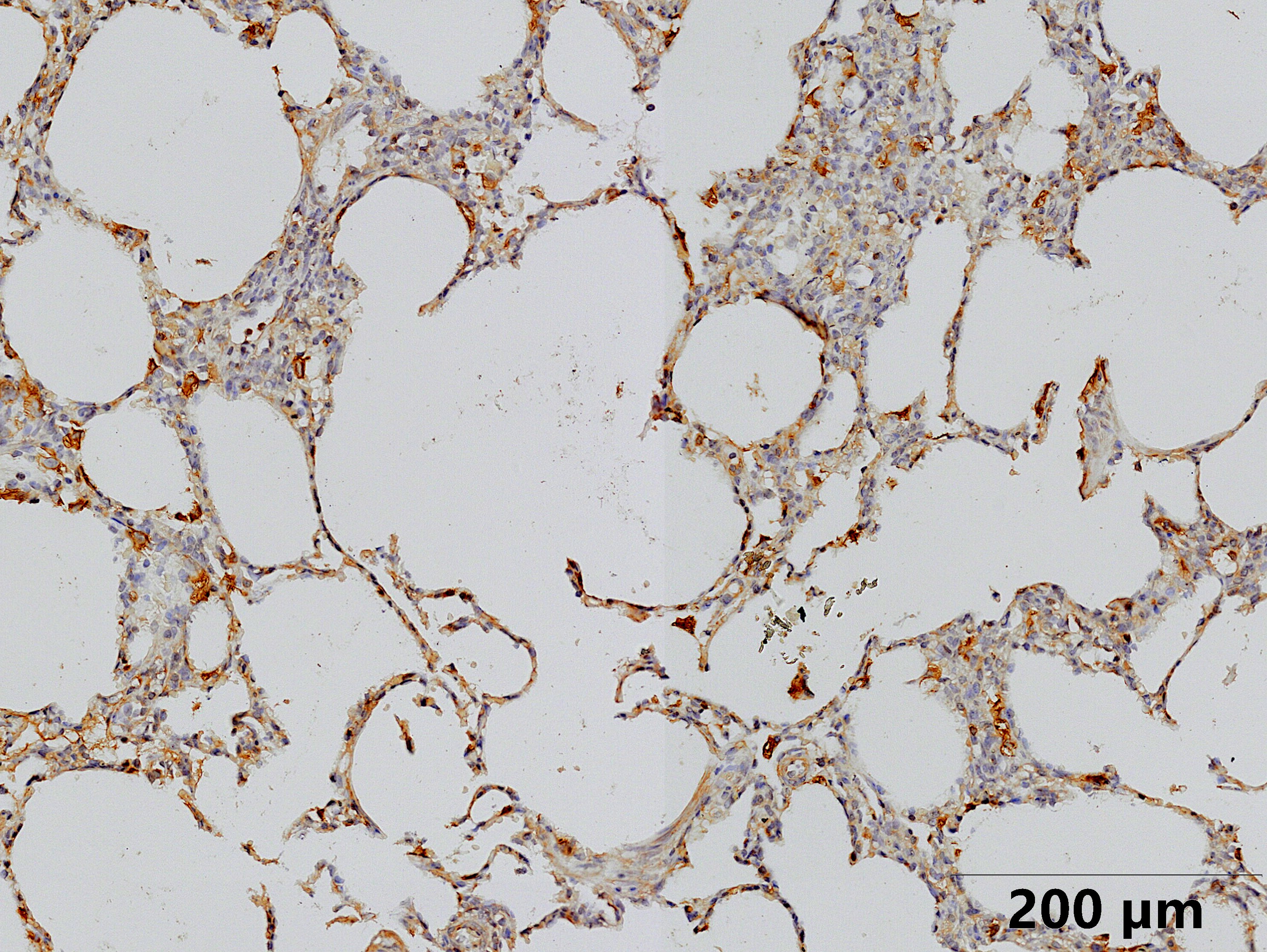

Supplement: Supplementary file 7 [file DataSheet7.ZIP › YY_IHC_Original image/Y1/图像_04.jpg]

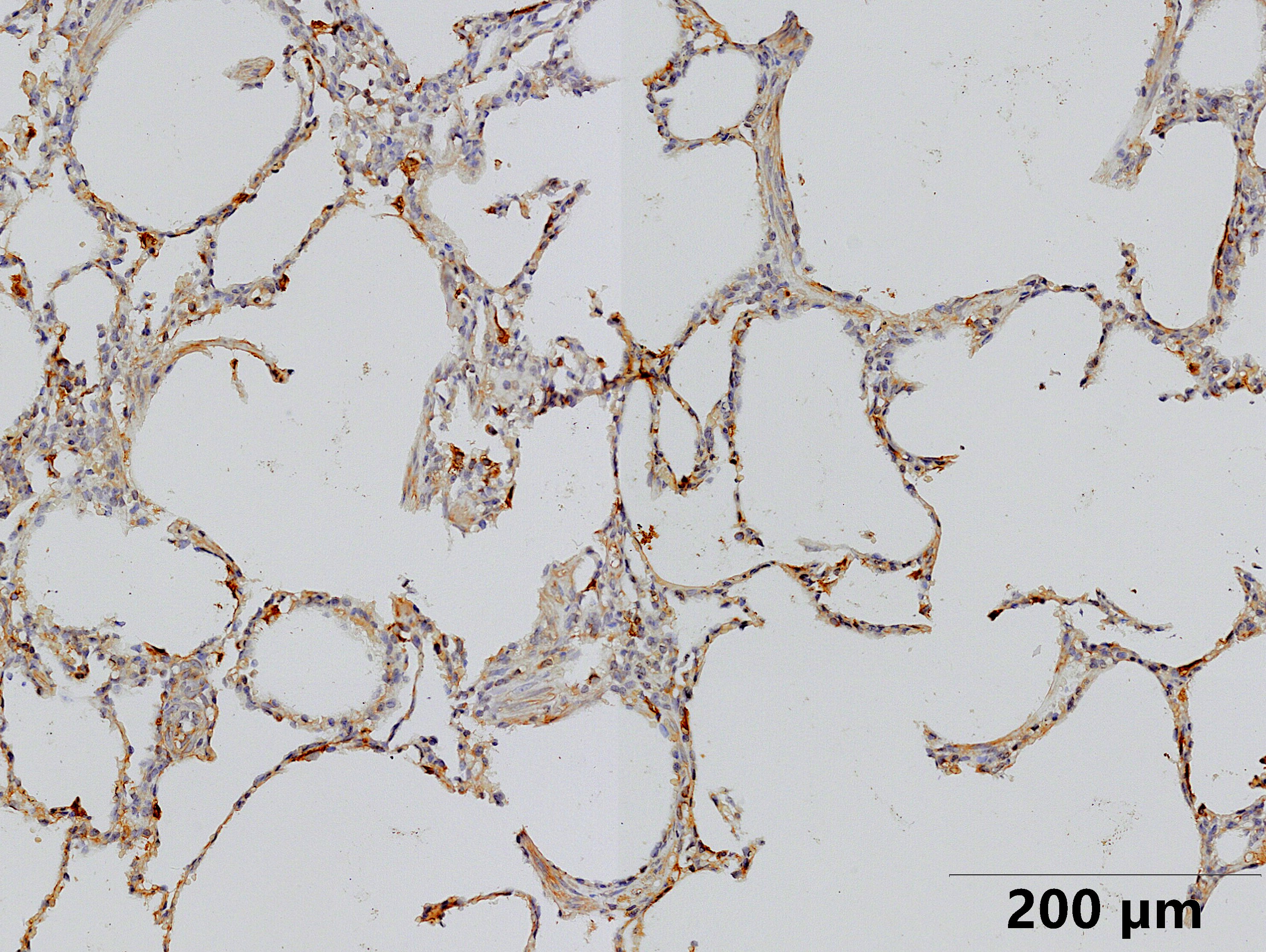

Supplement: Supplementary file 7 [file DataSheet7.ZIP › YY_IHC_Original image/Y1/图像_05.jpg]

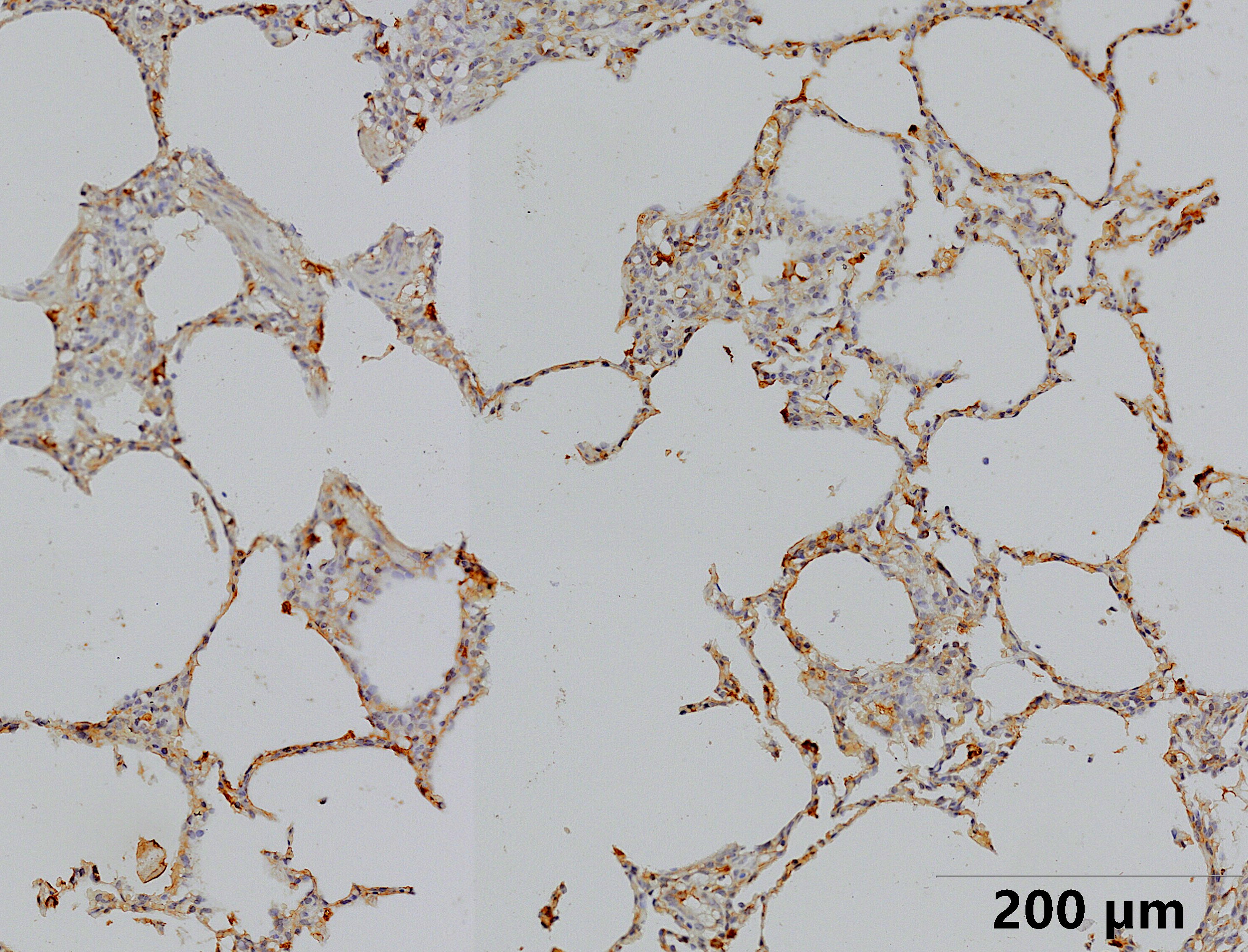

Supplement: Supplementary file 7 [file DataSheet7.ZIP › YY_IHC_Original image/Y2/图像_01.jpg]

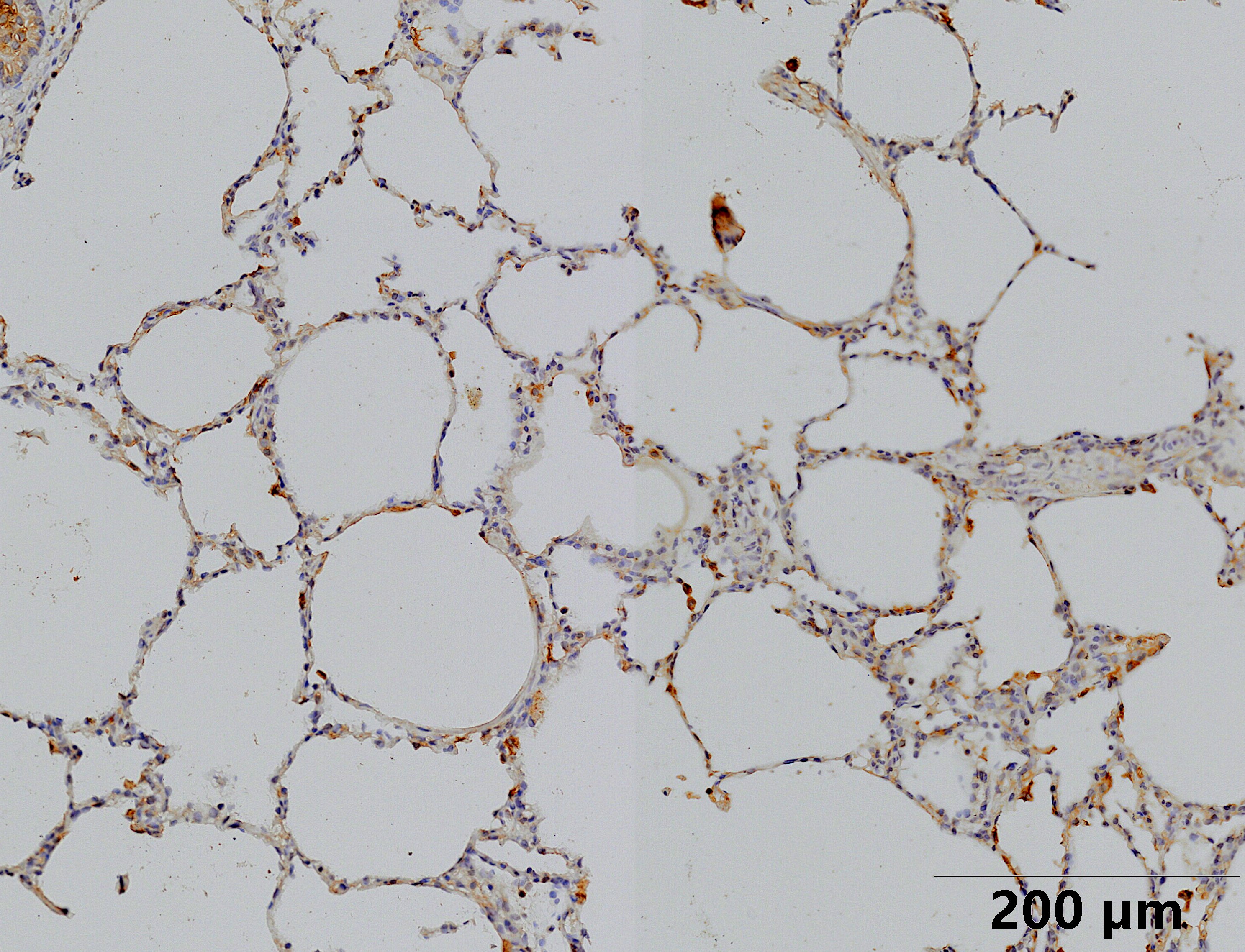

Supplement: Supplementary file 7 [file DataSheet7.ZIP › YY_IHC_Original image/Y2/图像_02.jpg]

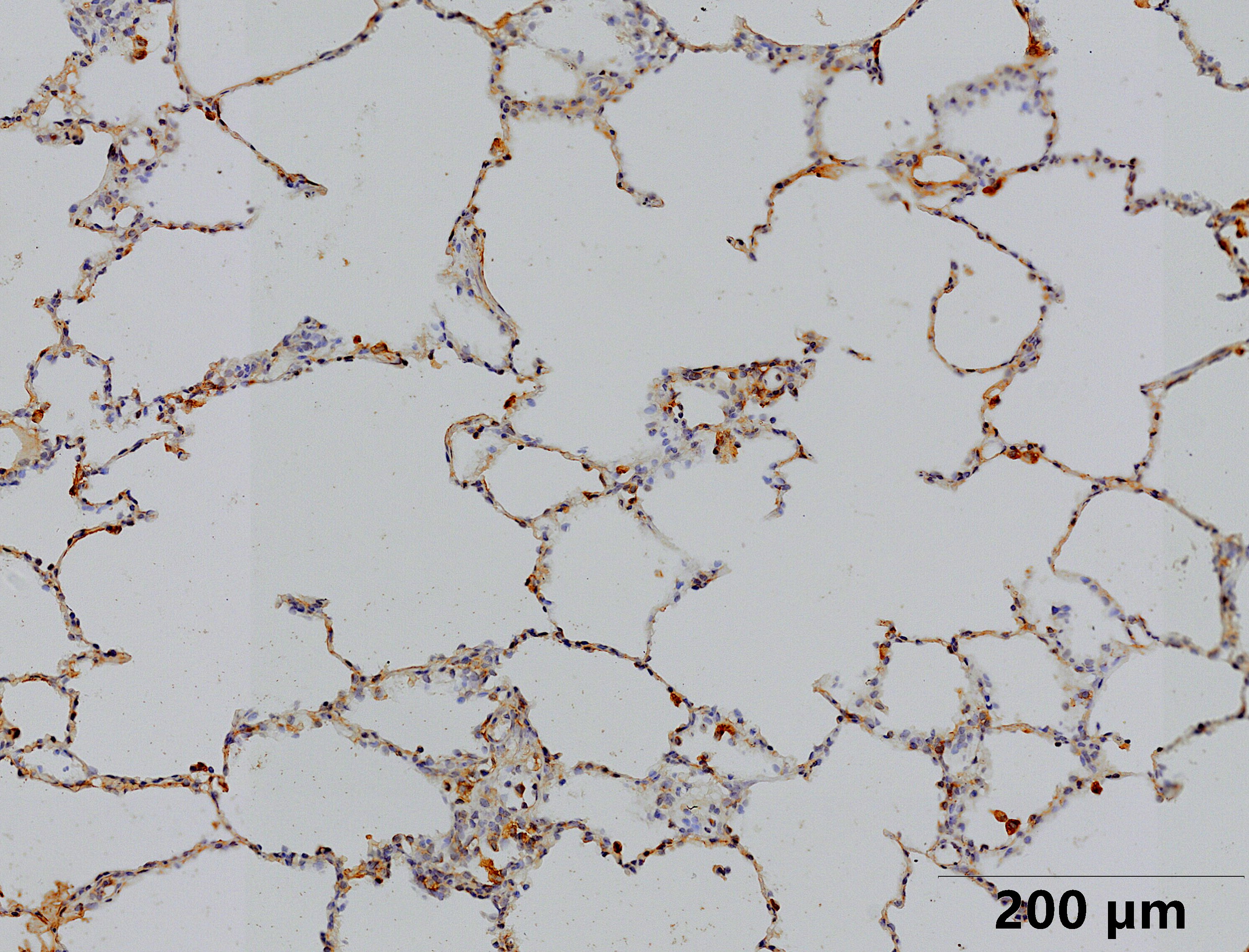

Supplement: Supplementary file 7 [file DataSheet7.ZIP › YY_IHC_Original image/Y2/图像_03.jpg]

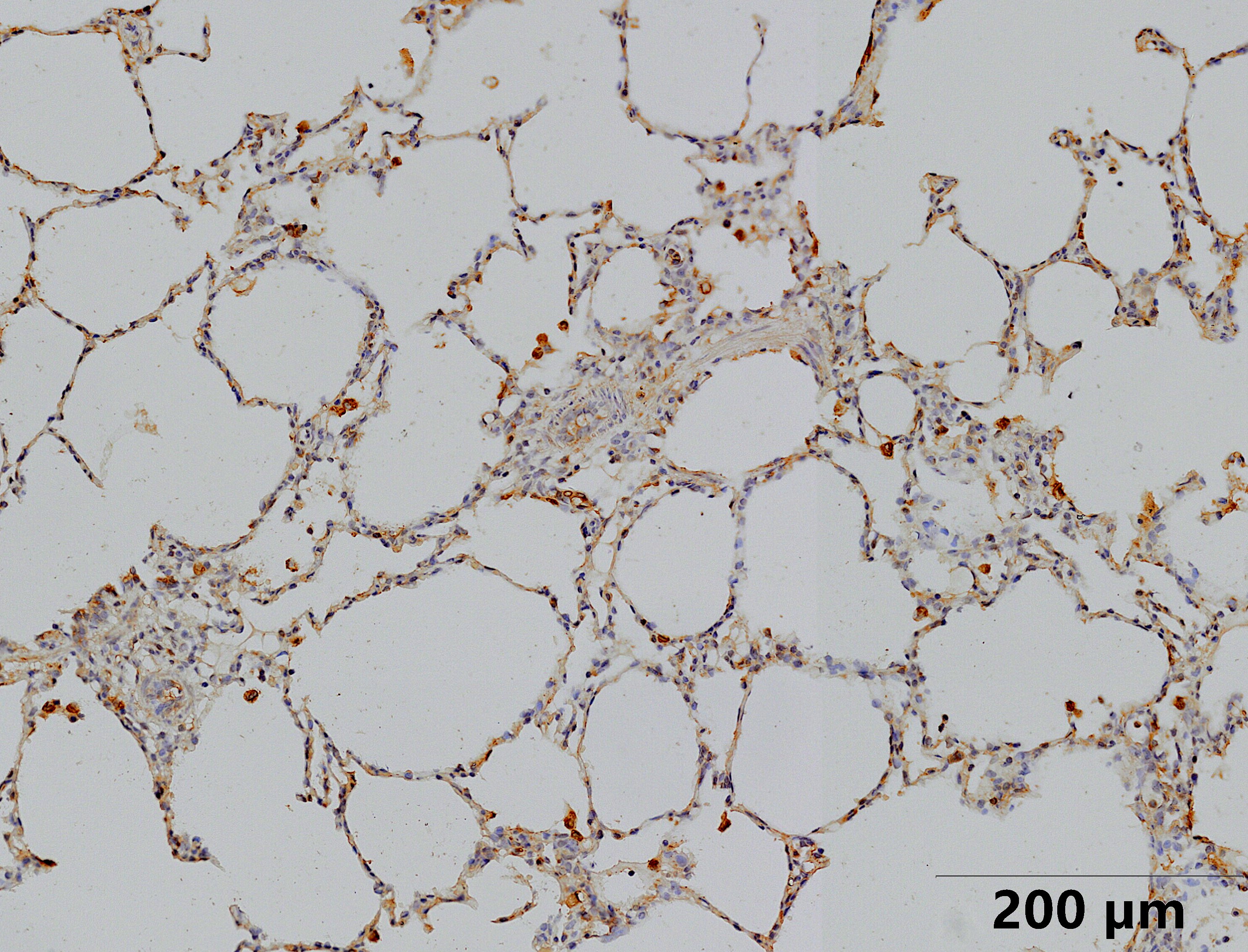

Supplement: Supplementary file 7 [file DataSheet7.ZIP › YY_IHC_Original image/Y2/图像_04.jpg]

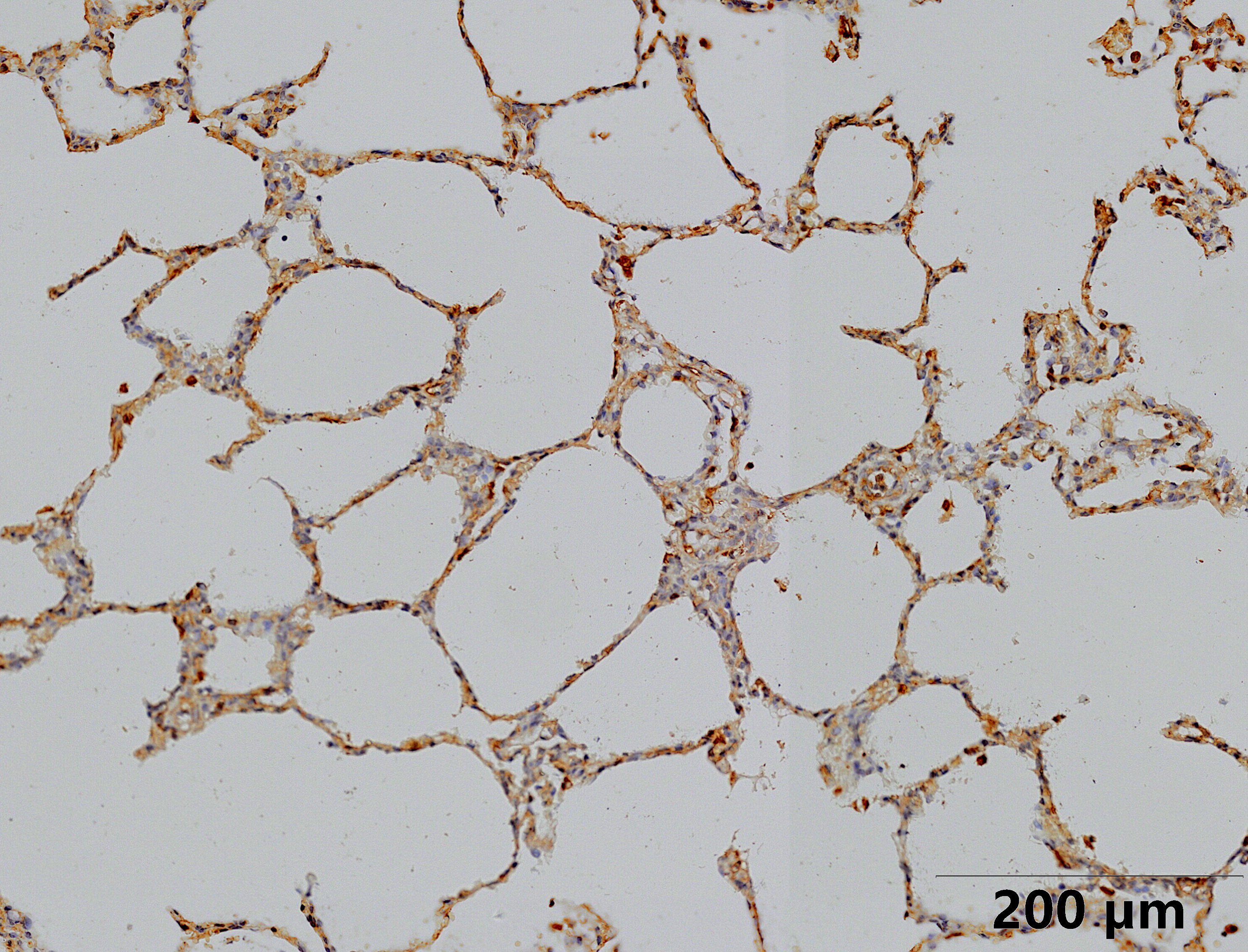

Supplement: Supplementary file 7 [file DataSheet7.ZIP › YY_IHC_Original image/Y2/图像_05.jpg]

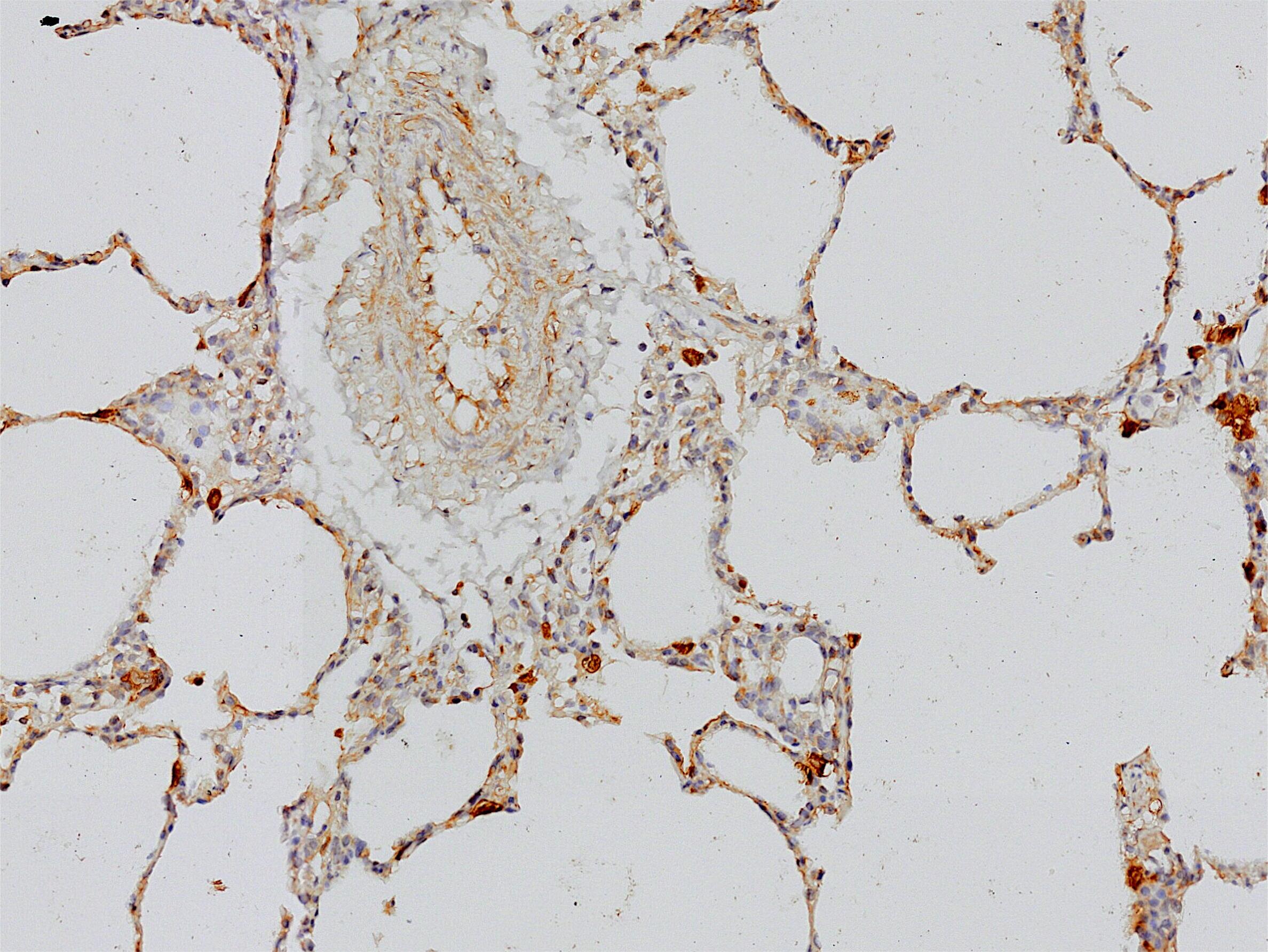

Supplement: Supplementary file 7 [file DataSheet7.ZIP › YY_IHC_Original image/Y3/图像_01.jpg]

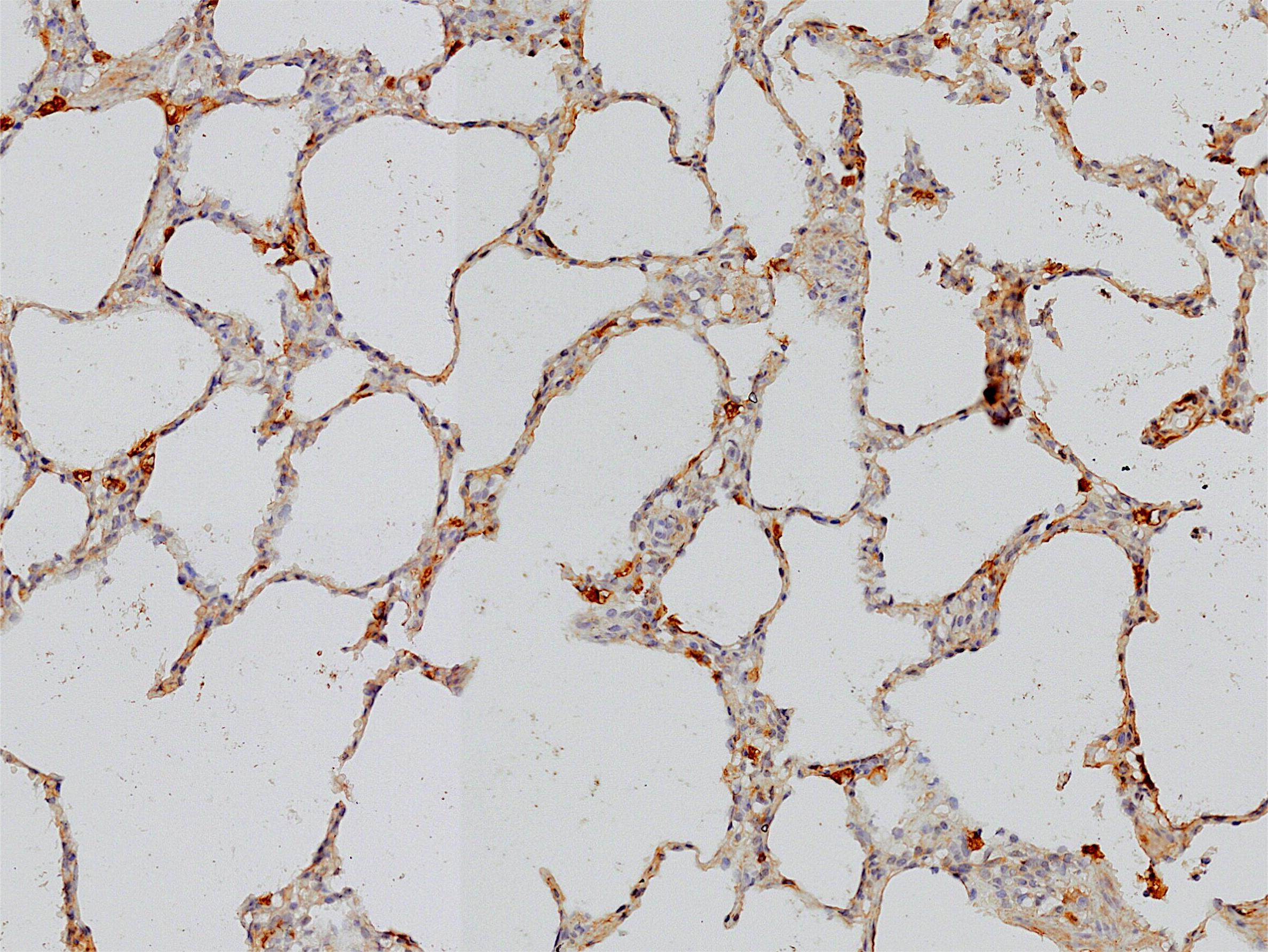

Supplement: Supplementary file 7 [file DataSheet7.ZIP › YY_IHC_Original image/Y3/图像_02.jpg]

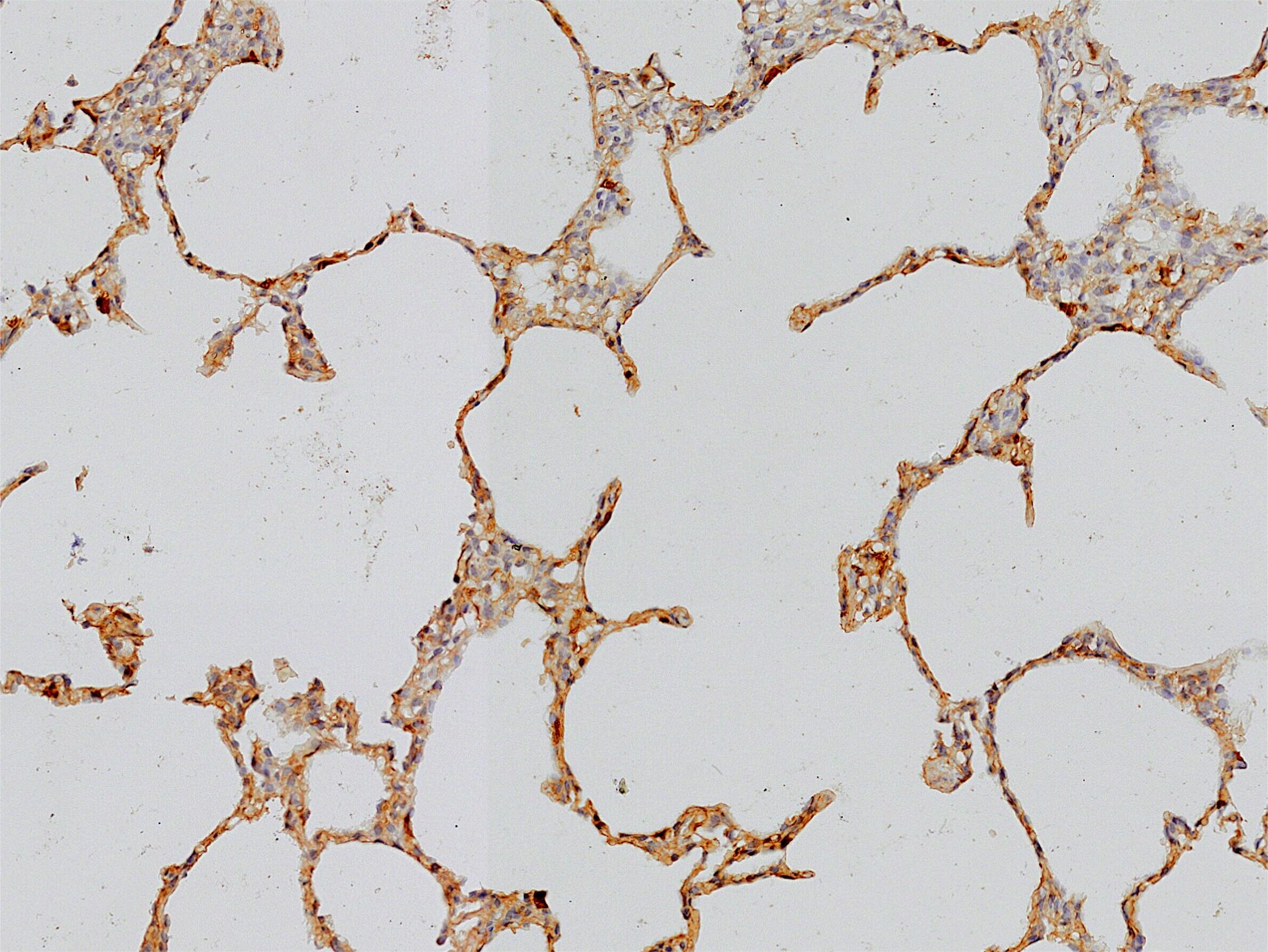

Supplement: Supplementary file 7 [file DataSheet7.ZIP › YY_IHC_Original image/Y3/图像_03.jpg]

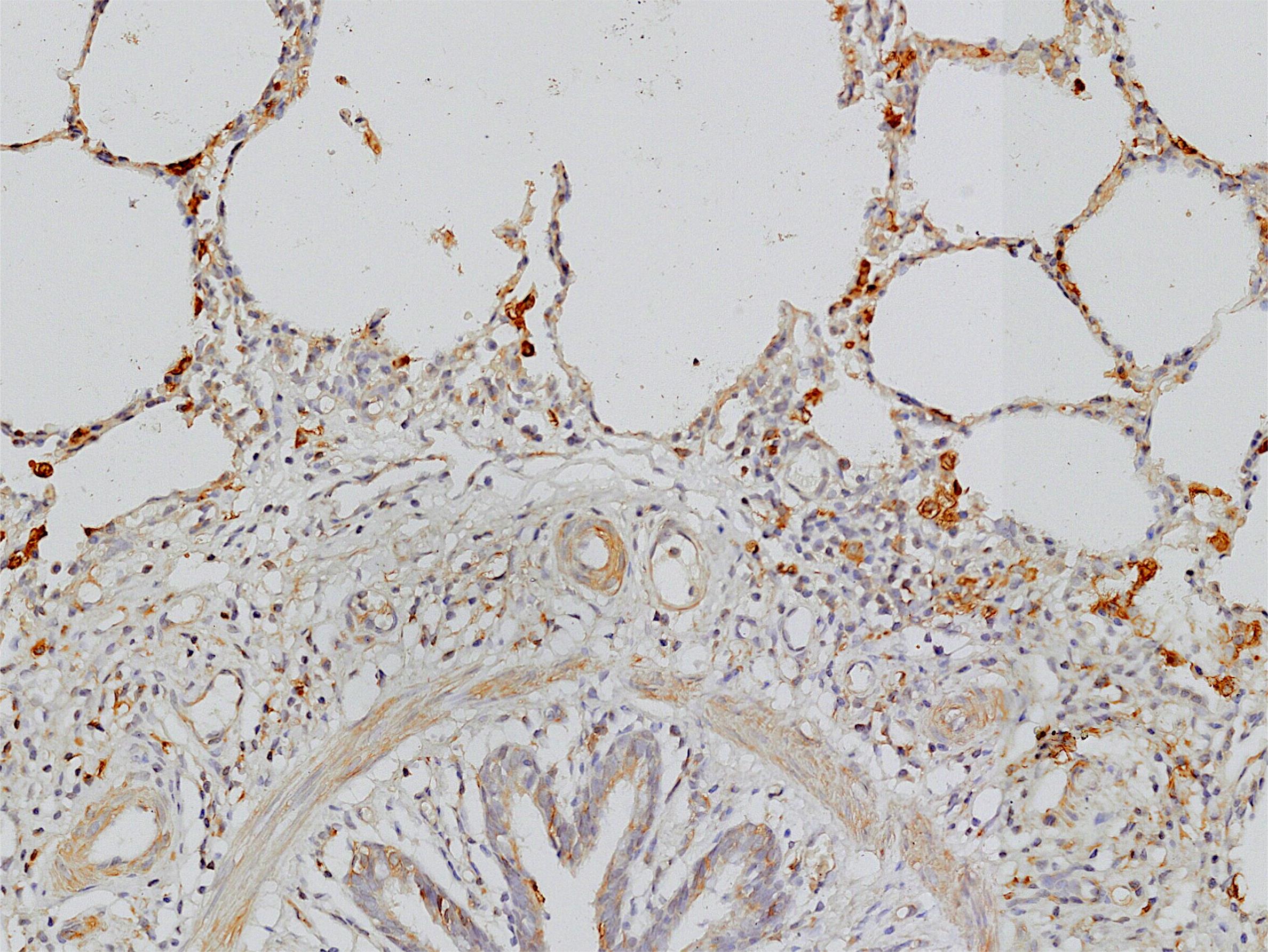

Supplement: Supplementary file 7 [file DataSheet7.ZIP › YY_IHC_Original image/Y3/图像_04.jpg]

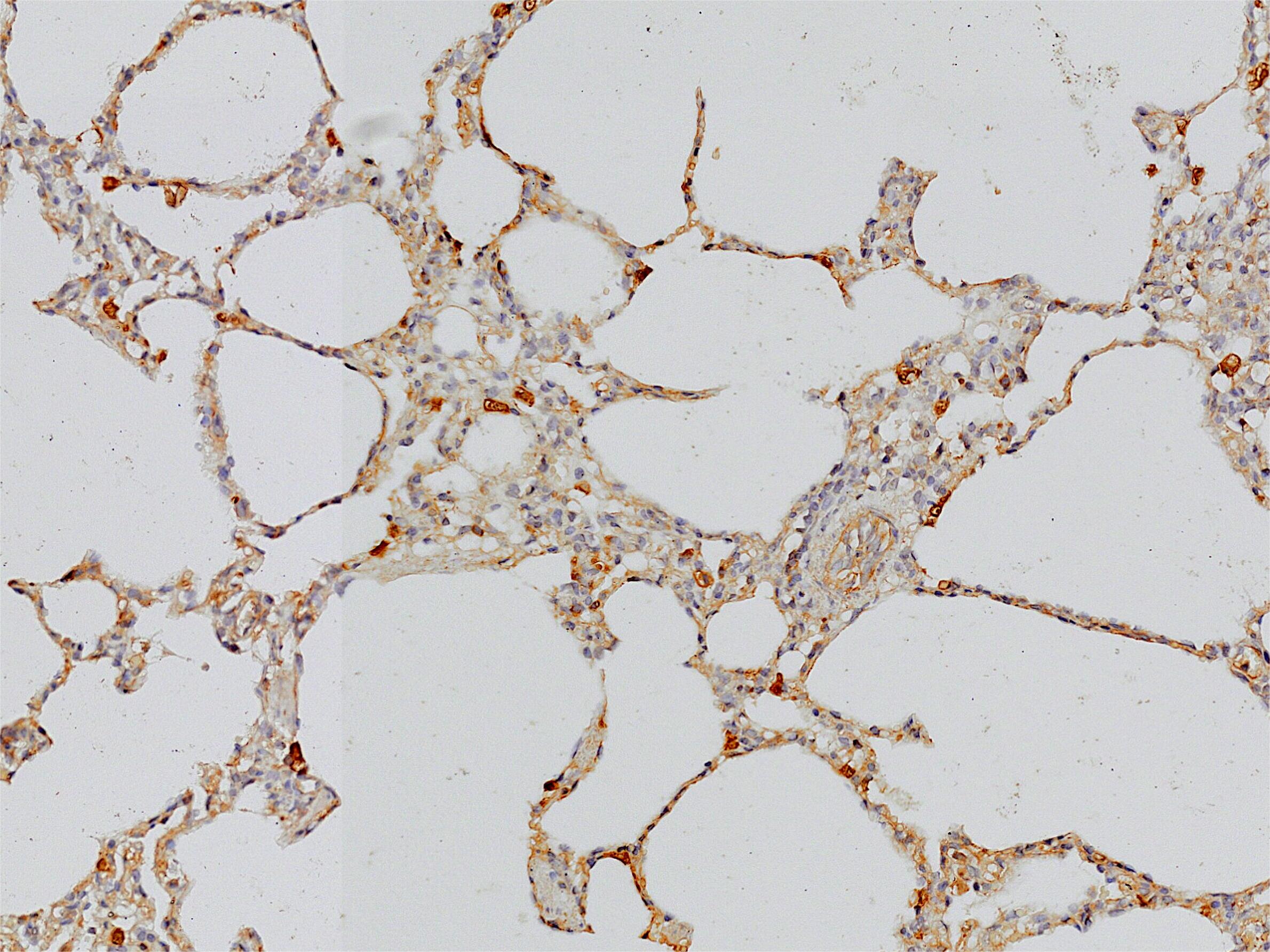

Supplement: Supplementary file 7 [file DataSheet7.ZIP › YY_IHC_Original image/Y3/图像_05.jpg]
